# Ammonia oxidation by novel “*Candidatus* Nitrosacidococcus urinae” is sensitive to process disturbances at low pH and to iron limitation at neutral pH^☆^

**DOI:** 10.1016/j.wroa.2022.100157

**Published:** 2022-10-04

**Authors:** Valentin Faust, Theo A. van Alen, Huub J.M. Op den Camp, Siegfried E. Vlaeminck, Ramon Ganigué, Nico Boon, Kai M. Udert

**Affiliations:** a Eawag, Swiss Federal Institute of Aquatic Science and Technology, 8600 Dübendorf, Switzerland; b ETH Zürich, Institute of Environmental Engineering, 8093 Zürich, Switzerland; c Department of Microbiology, RIBES, Radboud University Nijmegen, 0268 Nijmegen, The Netherlands; d Research Group of Sustainable Energy, Air and Water Technology, Department of Bioscience Engineering, Faculty of Science, University of Antwerp, 2020 Antwerpen, Belgium; e Centre for Advanced Process Technology for Urban Resource Recovery (CAPTURE), 9052 Gent, Belgium; f Center for Microbial Ecology and Technology (CMET), Faculty of Bioscience Engineering, Ghent University, 9000 Gent, Belgium

**Keywords:** Nitrification, Acidophilic AOB, Source separation, Chemical nitrite oxidation, Human urine, Life support system

## Abstract

Acid-tolerant ammonia-oxidizing bacteria (AOB) can open the door to new applications, such as partial nitritation at low pH. However, they can also be problematic because chemical nitrite oxidation occurs at low pH, leading to the release of harmful nitrogen oxide gases. In this publication, the role of acid-tolerant AOB in urine treatment was explored. On the one hand, the technical feasibility of ammonia oxidation under acidic conditions for source-separated urine with total nitrogen concentrations up to 3.5 g-N L^−1^ was investigated. On the other hand, the abundance and growth of acid-tolerant AOB at more neutral pH was explored. Under acidic conditions (pH of 5), ammonia oxidation rates of 500 mg-N L^−1^ d^−1^ and 10 g-N g-VSS^-1^ d^-1^ were observed, despite high concentrations of 15 mg-N L^−1^ of the AOB-inhibiting compound nitrous acid and low concentration of 0.04 mg-N L^−1^ of the substrate ammonia. However, ammonia oxidation under acidic conditions was very sensitive to process disturbances. Even short periods of less than 12 h without oxygen or without influent resulted in a complete cessation of ammonia oxidation with a recovery time of up to two months, which is a problem for low maintenance applications such as decentralized treatment. Furthermore, undesirable nitrogen losses of about 10% were observed. Under acidic conditions, a novel AOB strain was enriched with a relative abundance of up to 80%, for which the name “*Candidatus (Ca.)* Nitrosacidococcus urinae” is proposed. While *Nitrosacidococcus* members were present only to a small extent (0.004%) in urine nitrification reactors operated at pH values between 5.8 and 7, acid-tolerant AOB were always enriched during long periods without influent, resulting in an uncontrolled drop in pH to as low as 2.5. Long-term experiments at different pH values showed that the activity of “*Ca.* Nitrosacidococcus urinae” decreased strongly at a pH of 7, where they were also outcompeted by the acid-sensitive AOB *Nitrosomonas halophila*. The experiment results showed that the decreased activity of “*Ca.* Nitrosacidococcus urinae” correlated with the limited availability of dissolved iron at neutral pH.

## Highlights

• Novel acid-tolerant ammonia oxidizing bacteria “*Ca.* Nitrosacidococcus urinae” I8.
• Fast but unstable ammonia oxidation at low pH of real human urine.
• High nitrogen losses at low pH due to chemical nitrite oxidation.
• Decreasing activity of acid-tolerant ammonia oxidizing bacteria with increasing pH.

## Graphical abstract

**Figure:**
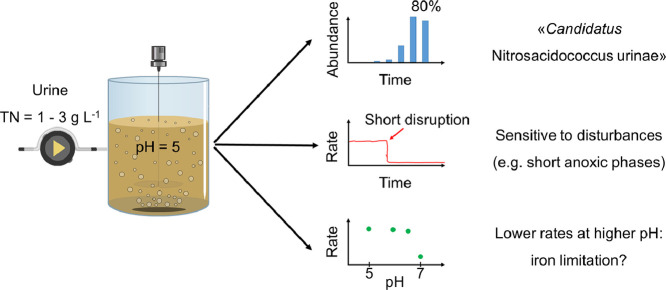

## 1 Introduction

Nitrification is an essential process in wastewater treatment in which ammonia (NH_3_) is biologically oxidized first to nitrite and then to nitrate (Tchobanoglous et al. 2014). Ammonia oxidation can be carried out by either ammonia-oxidizing bacteria (AOB), ammonia-oxidizing archaea (AOA), or bacteria performing complete ammonia oxidation (comammox), but AOB are dominant in wastewater treatment (Stein 2019). In conventional wastewater treatment, AOB such as *Nitrosomonas eutropha* are acid-sensitive and their activity declines with decreasing pH due to limited availability of their substrate NH_3_, which protonates to ammonium (NH_4_^+^), and ceases completely at a pH around 5.4 due to a direct pH effect related to the energy available from the proton motive force (Fumasoli et al. 2015). Nevertheless, AOB activity has been observed at pH substantially below 5.4 demonstrating the existence of acid-tolerant AOB (Fumasoli et al. 2017). Low pH values may occur when the alkalinity is insufficient to neutralize the protons released during ammonia oxidation. Examples of alkalinity-limited wastewaters include source-separated urine (Fumasoli et al. 2016), digester supernatant (Udert et al. 2008), and the effluent of chemically enhanced primary treatment with iron dosing (Taboada-Santos et al. 2020). Acid-tolerant AOB have been shown not only to survive at low pH values and have a high affinity for NH_3_ below 1 μg-N L^−1^ (Wang et al. 2021a), but also to withstand high free nitrous acid (HNO_2_) concentrations of more than 3 mg-N L^−1^ (Wang et al. 2021b). In contrast to AOB, no lower pH limit for nitrite-oxidizing bacteria (NOB) is reported. However, at low pH, NOB are often inhibited by HNO_2_, and chemical nitrite oxidation may become the dominant process for nitrite conversion (Udert et al. 2005).

Ammonia oxidation under acidic conditions has been tested for various applications. Acid-tolerant AOB were used by Li et al. (2020) as the first step of partial nitritation/anammox, in which partial nitritation was successfully achieved at pH values around 5 by suppressing NOB with HNO_2_ for highly diluted urine with total nitrogen (TN) concentrations of about 200 mg-N L^−1^. Since anammox bacteria are acid-sensitive, Li et al. (2020) suggested mixing the effluent with untreated urine to achieve a neutral pH. In a similar approach, Wang et al. (2021b) maintained partial nitritation at pH 4.5 to 5 using acid-tolerant AOB for a mixture of sewage with sidestream anaerobic digestion liquor with a TN concentration of about 100 mg-N L^−1^. Verhave et al. (2009) filed a patent concerning a process combining ammonia oxidation under acidic conditions and controlled chemical nitrite oxidation for the conversion of liquid manure into an ammonium-nitrate fertilizer. The patent application, however, was abandoned. Acid-tolerant AOB were also used to achieve self-sustaining HNO_2_ accumulation at low pH to enhance volatile solid destruction and nitrogen removal during aerobic digestion of waste activated sludge (Duan et al. 2019, Wang et al. 2021c). Last but not least, acid-tolerant AOB have been used for the bioconversion of methane to methanol at low pH (Zhang et al. 2021).

Even though ammonia oxidation under acidic conditions could open the door to new applications, the process can be problematic because during the chemical oxidation of nitrite harmful gases such as nitric oxide (NO), nitrogen dioxide (NO_2_), HNO_2_ and potentially N_2_O are released (Udert et al. 2005). One example from practice is an air biofilter from a pig stable, in which ammonia was found to be converted to nitrate at pH 2.5 by acid-tolerant AOB and chemical nitrite oxidation, but at the same time NO and NO_2_ gases were emitted (Picone et al. 2021). In urine nitrification, acid-tolerant AOB were a potential problem during long periods of low or no influent, as the pH dropped to values as low as 2.2 without alkalinity increase due to urine dosage. Such low pH resulted in nitrite instead of nitrate in the effluent and the release of harmful nitrogen oxide gases (Fumasoli et al. 2017). This is particularly dangerous for decentralized in-building settings or space applications such as the European Space Agency's Micro-Ecological Life Support System Alternative (MELiSSA) program where urine is supposed to be nitrified without the formation of harmful by-products (Clauwaert et al. 2017). In addition, the release of volatile nitrogen oxides results in nitrogen losses diminishing the potential for nutrient recovery.

In the above listed research projects about acid-tolerant AOB, different strains were found. While Li et al. (2020) and Fumasoli et al. (2016) observed the growth of *Nitrosospira* sp., Wang et al. (2021b) found a *Nitrosoglobus* sp. closely related to the non-halophilic “*Candidatus (Ca.)* Nitrosoglobus terrae” enriched from acidic tea soils (Hayatsu et al. 2017). In contrast, Picone et al. (2021) characterized a novel AOB with high identity to sequences found in reactors treating synthetic urine by Fumasoli et al. (2017) and proposed the name “*Ca.* Nitrosacidococcus tergens”. This bacterium was able to grow not only on ammonia but also on urea at pH values as low as 2.5.

At neutral pH, acid-tolerant AOBs are usually not abundant and acid-sensitive AOB dominate the nitrifying community. Wang et al. (2021a) argued that the acid-tolerant AOB *Nitrosoglobus* sp. are less competitive than acid-sensitive AOB such as *Nitrosomonas* sp. in pH-neutral wastewater treatment processes mainly due to their low maximum growth rate of 0.3 d^−1^ compared for example to 1.4 d^−1^ for *Nitrosomonas europaea*. Nevertheless, the effect of a more neutral pH (e.g. pH 7) on the activity of acid-tolerant AOB is unclear, and results from short-term experiments are contradictory: in Wang et al. (2021a), the activity was similar at pH 5 and 7, in Li et al. (2020), the activity increased with increasing pH value, and in Picone et al. (2021) the activity decreased at pH 7 compared to pH 5. These observations cannot be explained by substrate (NH_3_) limitation and product (HNO_2_) inhibition, which are common approaches used in activate sludge models (Sin et al. 2008). So far, the pH dependency of acid-tolerant AOB has only been determined with short-term experiments. No long-term experiments have been performed to investigate the activity of acid-tolerant AOB at neutral pH.

This publication addresses the role of acid-tolerant AOB in urine treatment and had two main objectives: (1) to investigate the technical suitability of ammonia oxidation under acidic conditions (pH 5) for source-separated urine with TN concentrations up to 3500 mg-N L^−1^; (2) to investigate the abundance and growth of acid-tolerant AOB at neutral pH values. A better understanding of the behavior of acid-tolerant AOB at neutral pH also helps to develop strategies to avoid the growth of acid-tolerant AOB. Specifically, the following four research questions were addressed:

• What are the most important AOB selected under acidic conditions and high TN concentrations?
• How well is acidic ammonia oxidation of source-separated urine suited for technical applications, especially with regard to process stability?
• How abundant are acid-tolerant AOB in urine nitrification reactors operated at pH values between 5.8 and 7?
• What is the long-term effect of neutral pH values on the activity of acid-tolerant AOB, and what is the underlying mechanism?

## 2 Materials and methods

### 2.1 Batch incubation

The transition from acid-sensitive to acid-tolerant AOB was investigated in several batch incubation experiments. For this purpose, activated sludge from the urine nitrification reactors at Eawag (Dübendorf, Switzerland), which were operated at pH values between 5.8 and 7 (Faust et al. 2022b), was added to an aerobic batch reactor without influent and pH control. Due to ammonia oxidation the pH dropped, and a pH decrease well below 5.4 indicated the growth of acid-tolerant AOB. The oxygen concentration was controlled between 4 and 6 mg L^−1^ with an on-off controller.

### 2.2 Setup and operation of the enrichment reactor

An ammonia oxidation reactor was operated under acidic conditions (pH of 5) for 700 days to enrich acid-tolerant AOB. An aerobic 12-L continuous-flow stirred-tank reactor (CSTR) without sludge retention was used to have a dynamic but simple system (more details are included in the supplementary information (SI) 1). The reactor was operated with suspended activated sludge, with an inlet at the bottom and an overflow at the top, through which effluent and activated sludge were discharged. To start up the reactor, activated sludge was added from an urine nitrification reactor at Eawag as described before (Section 2.1), but once the pH decreased to 4.9 due to ammonia oxidation, the influent was used to control the pH between 4.9 and 5 with an on-off controller. Stored source-separated urine, from waterless urinals and NoMix toilets (Gundlach et al. 2021), with low chemical oxidation demand (COD) was used as influent (pH ≈ 8.5). During storage, urea was fully hydrolyzed to ammoniacal nitrogen (Udert et al. 2006). To produce low COD influent a membrane-aerated biofilm reactor (MABR) was used, in which approximately 80% of the COD was degraded without nitrification. Separating COD degradation from nitrification allowed an enriched culture of AOB by avoiding high concentrations of heterotrophic bacteria. The temperature in the reactor was controlled at 25 °C, except for a period in summer when the temperature rose above 30 °C for two weeks due to very high outdoor temperatures. Dissolved oxygen (DO) was controlled between 4 and 6 mg L^−1^ using humidified air via an on-off controller.

### 2.3 Short-term respirometric activity tests

The effect of HNO_2_, pH, NH_3_, salinity, and DO, including anoxic conditions, on the activity of acid-tolerant AOB was evaluated with short-term activity experiments in a 3-L respirometer (set-up and experimental procedure in SI 2). The respirometer was operated either as two-chamber LSS respirometer (LSS: static gas, static liquid) or two-chamber LSF respirometer (LSF: static gas, flowing liquid) (van Loosdrecht et al. 2016). Activated sludge from the enrichment reactor was used for the activity tests. The pH in the respirometer was controlled with 0.4 M NaOH and 0.4 M HCl, and the temperature was set at 25 °C.

### 2.4 Long-term pH and iron experiments

The effects of pH and iron on acid-tolerant AOB were further investigated in two long-term experiments lasting 400 days and 70 days, respectively. In the first experiment, a 12-L reactor was operated as previously described (Section 2.2), and after 100 days the pH set-points were stepwise increased from 4.9/5 to 5.9/6, 6.4/6.5, and 6.9/7, with at least four weeks between changes. In the second experiment, a 12-L reactor was incubated with activated sludge from another acidic ammonia oxidation reactor, and after three weeks of operation, the pH set-points were increased from 4.9/5 to 6.9/7. Concomitant with the pH increase, FeCl_3_ (3 mg-Fe L^−1^) and the chelating agent nitrilotriacetic acid (NTA) were added to the reactor and the influent at a ratio of 1 mol-NTA mol-Fe^−1^. After another five weeks, the reactor was again fed with influent without iron addition.

To test whether acid-tolerant AOB were still abundant, 2 L of activated sludge were removed from the 12-L reactor, washed with nitrified urine, and added to an aerobic batch reactor without influent or pH control as described previously (Section 2.1). Again, a pH decrease well below 5.4 indicated the presence of acid-tolerant AOB.

### 2.5 Chemical nitrite oxidation model

A model was used to investigate whether the observed nitrate production could be explained by chemical nitrite oxidation processes. Kinetic and stoichiometric data of chemical nitrite oxidation were taken from Udert et al. (2005) and integrated in a Sumo2 model using the SUMO19 wastewater treatment software (Dynamita, France). The chemical nitrite oxidation consisted of the three nitrogen compounds equilibria and the chemical nitrite oxidation as shown in Eqs. (1) to (4). For completeness, the chemical oxidation of ammonia was also added (Eqs. (5) and (6)). All biological processes were turned off, and only chemical and physical processes were considered. The measured flow rates, the TAN (total ammoniacal nitrogen = NH_3_-N + NH_4_^+^-N) in the influent, the observed amount of ammonia oxidized to nitrite in the reactor (about 50% due to the limited alkalinity), and the measured pH in the reactor, were used as model inputs. Based on these input variables, the model calculated how much nitrite can be chemically converted to nitrate. More information as well as all equilibrium and rate constants can be found in the SI 3.

(1) $$NO_{2}^{-}+H^{+}↔\text{HN}O_{2}$$

(2) $$2\text{HN}O_{2}↔\text{NO}+NO_{2}+H_{2}O$$

(3) $$2\text{NO}+O_{2}\to 2NO_{2}$$

(4) $$2NO_{2}+H_{2}O↔\text{HN}O_{2}+NO_{3}^{-}+H^{+}$$

(5) $$N_{2}O_{3}\to \text{NO}+NO_{2}$$

(6) $$N_{2}O_{3}+NH_{3}\to N_{2}+\text{HN}O_{2}+H_{2}O$$

The goodness-of-fit between measured and simulated TNN (total nitrite nitrogen = HNO_2_-N + NO_2_^−^-N) and nitrate concentrations was evaluated with Eq. (7) using the model efficiency (E) according to Nash and Sutcliffe (1970),

(7) $$E=1-\;\frac{\sum_{i=1}^{n}{\left(y_{i}^{m}-y_{i}\right)}^{2}}{\sum_{i=1}^{n}{\left(y_{i}^{m}-{\bar{y}}_{m}\right)}^{2}}$$

where $y_{i}^{m}$ are the measured values and $y_{i}$ the corresponding simulated values of the i^th^ observation and ${\bar{y}}_{m}$ is the mean of all measured values. The closer the model efficiency is to the maximum of 1, the better the fit between measured values and simulation.

### 2.6 Chemical and physical analyses

Samples for the analyses of dissolved compounds were filtered through a 0.45 µm GF/PET filter (Chromafil, Macherey-Nagel). Cations (ammonium, sodium and potassium) and anions (nitrate, nitrite, chloride, phosphate and sulfate) were measured with ion chromatography (881 compact IC pro, Metrohm). The concentrations of trace elements including iron and copper were determined using inductively coupled plasma mass spectrometry (ICP-MS, Agilent 8900QQQ, Agilent). The acid-base equilibrium of ammonium and ammonia, and nitrous acid and nitrite were calculated according to Crittenden et al. (2012) using the dissociation constants of Anthonisen et al. (1976) corrected for ionic strength (Davies, 1967) (see SI 4 and SI 5). Dissolved COD in the influent was measured with photometric cuvette tests (LCK114, Hach Lange) using a spectrophotometer (DR 2800, Hach Lange GmbH). Nitrite and nitrate concentrations in the influent were measured with semi-quantitative colorimetric strips (110007 resp. 110020 MQuant, Merck). Total suspended solids (TSS) and volatile suspend solids (VSS) were measured according to the APHA (2012) standard protocol. Salinity was measured as conductivity with a standard conductivity cell (TetraCon 325, WTW), and pH was measured with a glass electrode (Orbisint CPS11D, Endress+Hauser). DO was measured with optical oxygen sensors (Oxymax COS61D and Memosens COS81D, Endress+Hauser).

### 2.7 Molecular analyses of the biomass

Biomass was sampled from the 12-L reactors and stored at -80 °C before further processing. Genomic DNA was extracted using the FastDNA Spin Kit for Soil (MP Biomedicals) with one modification to the manufacturer's protocol: to lyse the matrix, bead-beating steps (Bead Ruptor Elite, OMNI) were performed under conditions close to the MIDAS field guide (McIlroy et al. 2015) in series of 4 × 20 s at 6 m s^−1^ separated by 2 min on ice. The quality and concentration of the purified DNA extracts were assessed using NanoDrop Eight UV/Vis Spectrophotometer (Thermo Fischer Scientific Inc.) and Quibit 4 fluorometer (dsDNA assay kit, Thermo Fischer Scientific Inc.). DNA extracts were sent to LGC Genomics (Berlin, Germany) for 16S rRNA gene-based amplicon sequencing, library preparation and sequencing on an Illumina Miseq platform. The primer pair 341F (5’-CCTACGGGNGGCWGCAG-3’) / 785Rmod (5’-GACTACHVGGGTATCTAAKCC-3’) was used, targeting the V3-V4 hypervariable region of bacterial 16S rRNA gene sequences (Klindworth et al. 2013). To test for the presence of AOA, the primer pair 340F (5’-CCCTAYGGGGYGCASCAG-3’) / 1000R (5’-GGCCATGCACYWCYTCTC-3′) was nested with the universal primer pair U341F (5’-CCTAYGGGRBGCASCAG-3’) / U806R (5’-GGACTACGGGTATCTAAT-3’) and used to analyze the samples on day 49 and 273.

The data was processed with the mothur software package (v.1.40.5) (Schloss et al. 2009) as outlined by De Paepe et al. (2017). OTUs (Operational Taxonomic Units) were defined as a collection of sequences with a length between 393 and 429 nucleotides that were found to be more than 97% similar to one another in the V3-V4 region of their 16S rRNA gene after applying OptiClust clustering (Chen et al. 2013, Schloss and Westcott 2011, Schloss et al. 2009, Wang et al. 2012). Taxonomy was assigned using the Silva.nr_v138_1 database (Cole et al. 2014, Quast et al. 2013, Wang et al. 2007). The OTU table with taxonomy assignment was loaded into R, version 4.0.4 (2021-02-15), and singletons were removed (McMurdie and Holmes 2014, R Core Team 2016).

The extracted DNA samples after 259 days and 273 days of the enrichment reactor were additionally used for metagenome sequencing using the Illumina MiSeq and Oxford Nanopore platforms as described in Picone et al. (2021). Genome assembly was performed using NECAT (Chen et al. 2021) and quality of the assembly was checked using CheckM (Parks et al. 2015). Annotation was performed using Prokka (Seemann 2014). The complete genome sequence has been deposited in the NCBI BioProject database with accession number PRJEB52462.

## 3 Results and discussion

### 3.1 Reproducible growth of acid-tolerant AOB during long phases without pH control

**Fig. 1:**
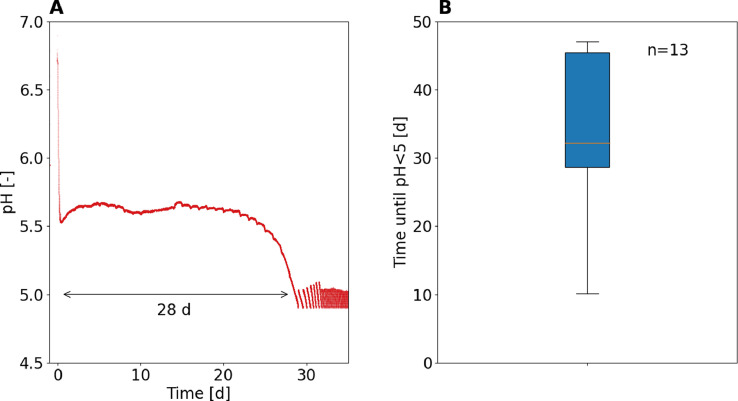
(A) pH evolution in the enrichment reactor as an example of a typical pH timeline. In this case, it took 28 days for the pH value to drop below 5. After reaching pH 5, the pH was controlled with the influent. (B) Boxplot of the time it took for the pH value to drop below 5 for 13 experiments with activated sludge from urine nitrification reactors. Details on the sludge origins and the pH timelines can be found in the SI 7.

In the 13 batch incubations without influent or base addition, the pH of activated sludge from urine nitrification reactors always dropped below 5 within 10 to 47 days, indicating reproducible growth of acid-tolerant AOB. As an example of a typical incubation experiment, Fig. 1A shows the pH timeline of the enrichment reactor. The pH decreased to about 5.5 within hours, where ammonia oxidation ceased and the pH remained or increased slightly. A similar pH limit of 5.4 was reported in Fumasoli et al. (2015) for partial nitrification of synthetic urine. The slight increase in pH during the idle phase could be related to CO_2_ volatilization. After 20 days, a second pH decrease was observed, which can be explained by microbial ammonia oxidation (Udert et al. 2005), indicating the growth of acid-tolerant AOB. When the pH had reached a value of 5 after 28 days, the enrichment reactor was controlled with the influent at pH 4.9 to 5. The start-up procedure was repeated using twelve sludge inocula from different urine nitrification reactors. While the pH values always decreased below 5, the time required varied strongly (Fig. 1B). No correlation was found between the time required for the pH to drop below 5 and different variables such as the operating pH before stopping the influent, the temperature or the VSS concentration (see SI 6 for scatterplot). Therefore, the different duration of growth cannot be explained with the available data. The long idle phase of at least 10 days between the first drop in pH to about 5.4 and the second drop in pH strongly suggests that the dominant AOB in all inocula were acid-sensitive and that the number of acid-tolerant AOB was low. When the pH was not controlled at around 5, the pH continued to decrease to values as low as 2.5 (see pH timeline of all inocula in SI 7), which has also been observed by Fumasoli et al. (2017).

### 3.2 Ammonia oxidation at low pH is fast but unstable

**Fig. 2:**
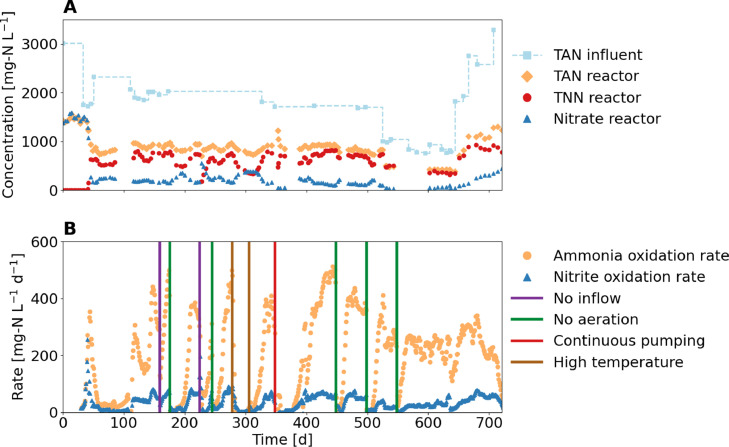
Performance of the enrichment reactor during more than 700 days. (A) Concentrations of the main dissolved nitrogen compounds in the reactor and influent: TAN = NH_4_^+^-N + NH_3_-N and TNN = NO_2_^−^-N + HNO_2_-N, (B) Measured ammonia and nitrite oxidation rates and operational disturbances (vertical lines). All additional measured variables as well as the HNO_2_ and NH_3_ concentrations can be found in the SI 8. The temperature was controlled at 25 °C except for the indicated period with high temperatures above 30 °C.

In the enrichment reactor, ammonia oxidation rates with a maximum of about 500 mg-N L^−1^ d^-1^ and specific rates of 10 g-N g-VSS^-1^ d^-1^ were observed at 25 °C, despite high HNO_2_ concentrations of 15 mg-N L^-1^ and low NH_3_ concentrations of 0.04 mg-N L^−1^ (Fig. 2). At such high HNO_2_ and low NH_3_ concentrations, the activity of the most common AOB in wastewater treatment is severely reduced (Sin et al. 2008). The maximum rates are of the same order of magnitude as those previously found for nitrification at pH 5.8 to 6 (Fumasoli et al. 2016), where rates between 120 to 640 mg-N L^-1^ d^-1^ were reported for a moving bed biofilm reactor.

Four different types of operational disturbances occurred (Fig. 2B), resulting in sharp drops in the ammonia oxidation rates (see SI 9 for details about the operational disturbances): (i) Influent stop and thus no pH control resulted in a pH drop to values as low as 4 and increased HNO_2_ concentrations. (ii) Interruption of aeration led to anoxic conditions in the reactor during 3 to 15 h. (iii) Uncontrolled continuous pumping of around 12 L, equivalent to one reactor volume, for 12 h resulted in a pH increase to as high as 8.5 and biomass washout. (iv) Insufficient temperature control resulted in temperatures as high as 33 °C. The system always recovered, but it took up to two months to reach the ammonia oxidation rate the system had before the operational disturbances. This is not a system artifact, as the acidic reactor, in which the “*Ca.* Nitrosacidococcus tergens” dominated was also very sensitive to short periods without oxygen or substrate supply (H.J.M op den Camp, personal communication, 15.01.2022). Around day 60 and day 700, the ammonia oxidation rate also decreased, although no obvious process control error occurred. However, the failure after 700 days might be linked to an increase in the influent concentration due to a change of the influent tank. In other acidic ammonia oxidation systems where the solid retention time (SRT) was controlled with a membrane bioreactor, such large fluctuations in ammonia oxidation rates were not observed (Picone et al. 2021, Wang et al. 2021b). Therefore, controlling the SRT could lead to a more stable ammonia oxidation rate. Nevertheless, process disturbances such as lack of aeration or influent would still result in high activity losses with long recovery times. Overcoming such obstacles would require detailed planning, e.g., to avoid running out of urine, frequent maintenance, e.g., to avoid pump failure, and very careful operation.

### 3.3 Chemical nitrite oxidation caused high gaseous nitrogen losses

Nitrate in the enrichment reactor was mainly produced by chemical nitrite oxidation. Except for a first peak observed after 40 days, nitrite oxidation rates were below 80 mg-N L^−1^ d^−1^ throughout the 700-day experiment (Fig. 2). Most likely, the first peak was caused by biological nitrite oxidation, but once the HNO_2_ concentration exceeded 0.5 mg-N L^−1^ around day 42, the biological rate decreased and chemical nitrite oxidation became dominant, as common NOB are strongly inhibited already at HNO_2_ concentrations of 0.1 mg-N L^−1^ (Sin et al. 2008). The nitrite accumulation ratio (NAR), which is the amount of nitrite not converted to nitrate, was on average 77%, excluding the start-up phase (timeline of NAR in SI 10). During periods with hydraulic retention times (HRT) of less than 2 days, NAR increased to 94%. TAN and the sum of TNN and nitrate-N were in a ratio of about 1:1, which was expected due to the limited alkalinity of urine (Fumasoli et al. 2016). Dissolved nitrogen losses were 11% of the nitrogen in the influent (see SI 11 for timeline). For comparison, in urine nitrification at pH values between 5.8 and 6 and low HNO_2_ concentrations, Fumasoli et al. (2016) reported that nitrogen losses were negligible. Li et al. (2020) also reported lower nitrogen losses of up to 4% for partial nitritation under acidic conditions. Since nitrogen was most likely lost as gaseous NO and NO_2_ (Fumasoli et al. 2017), the higher chemical nitrite oxidation rate due to higher HNO_2_ concentrations (see next paragraph) in this research project may explain the observed difference. Fumasoli et al. (2017), on the other hand, reported higher nitrogen losses of about 50% at pH 2.5, which most likely was related to the high HRT of 88 days.

**Fig. 3:**
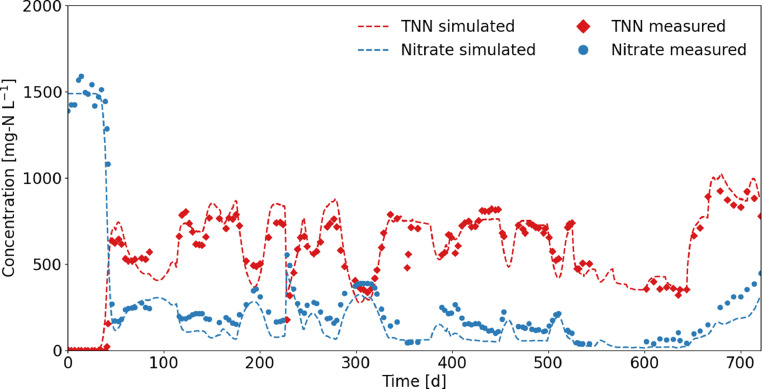
Measured and simulated TNN (= NO_2_^−^-N + HNO_2_-N) and nitrate concentrations in the reactor. The model simulated the chemical nitrite oxidation and did not consider biological processes.

The chemical nitrite oxidation model predicted the TNN and nitrate concentrations well, with model efficiencies of 0.88 and 0.94, respectively (Fig. 3). Only the first peak of nitrite oxidation around day 40 was not well captured by chemical nitrite oxidation (see SI 12). This confirms that biological nitrite oxidation was initially observed, but with increasing HNO_2_ concentrations and decreasing HRT, NOB are washed out and chemical nitrite oxidation became dominant. An operational failure that resulted in a drop in pH to 4 and increased chemical nitrite oxidation on day 226 was also well captured by the model. According to the model, the chemical nitrite oxidation rate increases with increasing HNO_2_ and DO concentrations in the reactor (see simulations in SI 13). Zuo et al. (2022) used the chemical nitrite oxidation model with the same rate constants and also obtained a good fit, but this is the first time the model has been used for continuous operation.

### 3.4 High abundance of novel “*Candidatus* Nitrosacidococcus urinae” at low pH

**Fig. 4:**
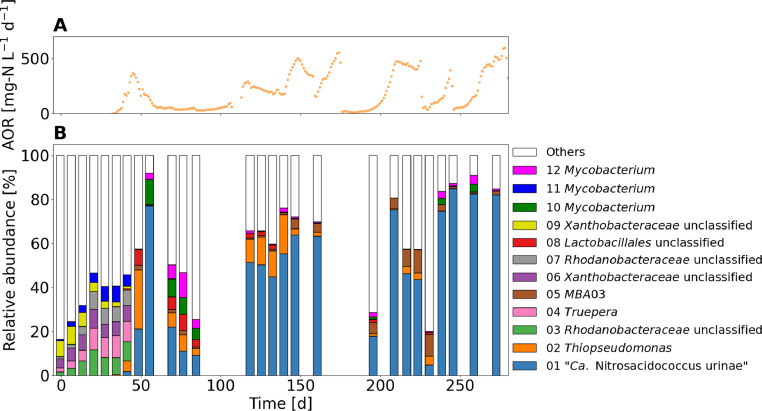
(A) Ammonia oxidation rate (AOR). (B) Microbial community composition at genus level (top 12 OTUs) of the enrichment reactor at pH 5 fed with source-separated urine. Biomass was only sequenced for the first 300 days, as this should give a representative picture of the community and its dynamics.

An acid-tolerant AOB of the genus *Ca.* Nitrosacidococcus was enriched with a relative read abundance of up to 80% (Fig. 4). The dominant AOB in the inoculum, OTU 38 *Nitrosomonas* sp., was closely related to the *N. europaea* lineage and had a relative read abundance of approximately 1% (see SI 14 for abundance of AOB). *N. europaea* was also previously found to be the dominant AOB lineage in partial urine nitrification (Fumasoli et al. 2016). Between days 35 and 42, the abundance of *Nitrosomonas* sp. decreased rapidly and OTU 01 emerged that had 99.3% gene identity with the acid-tolerant AOB “*Ca.* Nitrosacidococcus tergens” RJ19 (see SI 15 for phylogenetic tree). "*Ca.* Nitrosacidococcus urinae" was not detectable in the inoculum but a closely related *Ca.* Nitrosacidococcus sp. (99.5% gene identity) was found at a low abundance of 0.004%. Fluctuations in the relative abundance of *Ca.* Nitrosacidococcus sp. appeared to be related to the variations in ammonia oxidation rates as can be seen when comparing Fig. 4A and B. No AOA were found in the analyzed samples.

The last two samples (259 days and 273 days) were used for metagenome sequencing. Due to the high enrichment level, a complete circular genome of a novel *Ca.* Nitrosacidococcu*s* sp. was obtained with an average nucleotide identity (ANI) of 87.15% compared to “*Ca.* Nitrosacidococcu*s* tergens” (Picone et al. 2021) and only 75.04% compared to "*Ca.* Nitrosoglobus terrae" (Hayatsu et al. 2017). The ANI identity value was below the cut-off of 95% for species delimitation, and therefore the name “*Candidatus* Nitrosacidococcus urinae” I8 is proposed for this novel acid-tolerant ammonia oxidizer, named after the urine medium it was enriched on. The circular genome of strain I8 consisted of 1’848’551 bp, and contained 1739 protein coding sequences, two full rRNA operons, 44 tRNA's, 1 tmRNA, and 2 repeat regions. Genes encoding the major enzymes involved in ammonia oxidation, urea utilization, energy conservation, carbon fixation (CBB cycle), glycolysis and gluconeogenesis were present and overall the genome was highly comparable to that of “*Ca.* Nitrosacidococcus tergens” (Picone et al. 2021), which also lacked a soluble cytochrome c554 (CycA), a NO-producing nitrite reductase (NirK), and the canonical AmtB-type transporters for ammonia uptake. Further, they both lack a siderophore system. No nitrite oxidoreductase (NXR) genes were found in the metagenome sequence, thus no NOB or comammox species were present.

Although the inoculum had high biological nitrite oxidation rates, the amplicon data are not conclusive for NOB. While OTU 06 *Xanthobacteraceae* unclassified, had a high identity with *Nitrobacter* sp. 219, none of the *Nitrobacter* and *Xanthobacteraceae* linked OTUs clustered with known NOB (see SI 16 for phylogenetic tree). It should be taken into account that the V3-V4 sequences of this bacterial group do not allow firm conclusions. Considering the high dominance of OTU 06 and OTU 09 (together 11%) NXR genes or metagenome sequence sequencing of the DNA extracted from the inoculum could be interesting. Regardless, the relative abundance of all *Nitrobacter* and *Xanthobacteraceae* linked OTUs that were considered potential NOB decreased below 0.1% after 42 days (see SI 16 for the relative abundance of *Nitrobacter* and *Xanthobacteraceae* linked OTUs).

The shift in the microbial community in general around day 42 was also reflected in the decrease in microbial diversity (see SI 17 for boxplot), which may be related to selective conditions at pH 5 and the use of COD pretreated influent. The COD pretreatment step resulted in an 80% lower COD concentration in the influent compared to the influent of the original nitrification reactor, and thus less heterotrophic biomass. The VSS concentration in the reactor decreased from 1000 mg-VSS L^−1^ to 200 mg-VSS L^−1^ within the first 50 days (timeline in SI 8). The particle size distribution showed that no large aggregates (diameter > 100 µm) were formed (particle size distribution in SI 18). Therefore, a pH adaption mechanism using pH-neutral microenvironments such as granules or biofilm (De Boer and Kowalchuk 2001) can be excluded in the present system.

### 3.5 Kinetic aspects of “*Candidatus* Nitrosacidococcus urinae” I8

#### 3.5.1 Net growth rate at pH 5

Since no sludge retention was applied, the HRT was equal to the solid retention time (SRT). From the minimum observed SRT of 1.6 d, a maximum net growth rate of 0.6 d^−1^ was estimated at pH 5 and 25 °C (SI 19). This is higher than the maximum net growth rates of 0.18 d^−1^ and 0.2 d^−1^ reported for *Ca.* Nitrosoglobus sp. at pH 5 and 22 °C (Wang et al. 2021a) and “*Ca.* Nitrosacidococcus tergens” at pH 3.5 and 22 °C (Picone et al. 2021), respectively.

#### 3.5.2 Short-term activity tests confirm high sensitivity of acidic ammonia oxidation

**Fig. 5:**
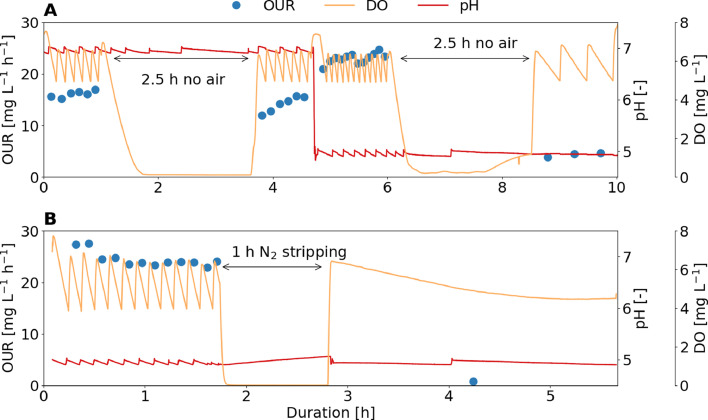
Influence of anoxic conditions on the activity expressed as the oxygen uptake rate (OUR) of activated sludge dominated by “Ca. Nitrosacidococcus urinae” I8. (A) 2.5 hours of non-aerated phases at pH 5 and 7. (B) 1 hour of supplying N_2_ gas instead of air at a pH 5.

While a non-aerated phase of 2.5 hours at neutral pH had no lasting effect on the activity of the sludge dominated by acid-tolerant AOB, interrupting aeration at low pH caused a sharp drop of the activity by 90% (Fig. 5A). This implies that the non-aerated phase is only a problem at low pH, and that the cause of the inhibitory effect must be related to the acidic conditions, for instance, a product of a biological or chemical reaction that only occurs at low pH, such as chemical nitrite oxidation. Due to chemical nitrite oxidation, the NO concentration increases during non-aerated phases (see the simulation in SI 20). Because NO is a known biocide (Shank et al. 1962), it was suspected that the decrease in activity after anoxic conditions was related to the accumulation of NO. However, the stripping of gaseous products including NO with N_2_ gas for one hour resulted in a complete cessation of ammonia oxidation, which means that negative effects by NO are at least not the only reason for the activity decrease at low pH values (Fig. 5B and simulation in SI 21). Instead, the harsh conditions at low pH (e.g. acid pH and high HNO_2_ concentration) may require continuous energy production to sustain the pH homeostasis mechanism (Krulwich et al. 2011), which cannot be maintained during phases with low oxygen concentrations. This effect might be further aggravated by the presence of NO, which is known to react with the respiratory chain and inhibit oxygen respiration (Zhou et al. 2011).

**Fig. 6:**
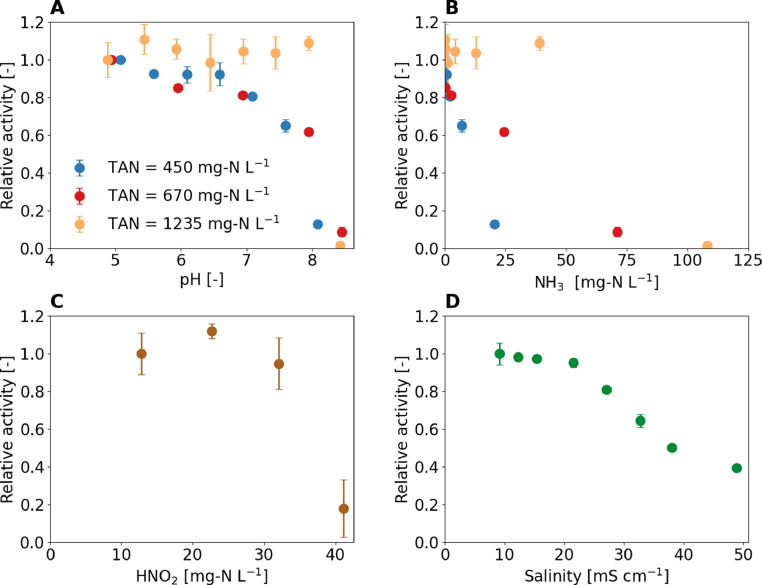
Influence of different environmental conditions on the activity of activated sludge dominated by “Ca. Nitrosacidococcus urinae” I8. The activity is expressed as relative activity by dividing the oxygen uptake rate (OUR) through the OUR without changes at pH 5. (A) pH (B) NH_3_, (C) HNO_2_, and (D) salinity. (A) and (B) are from the same experiment.

Besides oxygen deficiency, increasing the pH above 8 also resulted in an activity drop (Fig. 6A). NH_3_ concentrations during the pH experiments indicated that the activity decrease was not due to inhibition by NH_3_ (Fig. 6B). For example, in the experiment with TAN of 450 mg-N L^−1^ the activity dropped to 10% when the NH_3_ concentration was 20 mg-N L^−1^ and in the experiment with TAN of 1235 mg-N L^-1^ no activity was lost despite NH_3_ concentrations of 40 mg-N L^−1^. The strong pH effect above 8 could explain why an operational failure leading to continuous pumping on day 348 causing a pH increase to around 8.5 had a long lasting effect on the ammonia oxidation rate.

The acid-tolerant AOB could withstand very high HNO_2_ concentrations of up to 30 mg-N L^−1^, but at a concentration above 40 mg-N L^−1^, the activity dropped sharply (Fig. 6C). For common AOB in wastewater treatment, HNO_2_ half inhibition constants of 0.07 to 2.8 mg-N L^−1^ are reported (Sin et al. 2008), which means that they are inhibited by 91% to 99% at HNO_2_ concentrations of 30 mg-N L^-1^. The drop in activity at concentrations above 40 mg-N L^−1^ could explain the cessation of ammonia oxidation following a reactor operational failure without influent, such as on day 225. At a nitrite concentration of 500 mg-N L^−1^, a pH drop below 4.2 would already lead to a HNO_2_ concentration above 40 mg-N L^−1^. Batch experiments without pH control were performed with activated sludge from the main enrichment reactor to which either nitrite or ammonium was added. The different TAN concentrations did not affect the pH, at which the ammonia oxidation ceased. However, different TNN concentrations of 350 mg-N L^-1^, 700 mg-N L^−1^, and 1200 mg-N L^−1^ did affect the minimum pH values reached. This observation suggests that the cessation of ammonia oxidation at low pH is most likely not related to NH_3_ or a direct pH effect, but rather to the HNO_2_ concentration (see batch experiments in SI 22). It is reported that HNO_2_ not only inhibits bacteria but is irreversibly toxic (Zhou et al. 2011), explaining why the activity in the reactor was not immediately recovered once the pH was increased and therefore the HNO_2_ concentration decreased again. Another possibility is that due to enhanced chemical nitrite oxidation at higher HNO_2_ concentrations, the acid-tolerant AOB are killed by the increased NO concentration (simulation in SI 23) and not by the HNO_2_ directly. Direct NO measurements would be required to further investigate the role of NO.

At values above 20 mS cm^−1^, salinity had an inhibitory effect on the activity of acid-tolerant AOB (Fig. 6D), which is consistent with what was found for acid-sensitive AOB in urine treatment (own data, Faust et al. 2022a) and SI 24). Since the acid-tolerant AOB *Nitrosoglobus* sp. is more sensitive to salinity Wang et al. 2021a), salinity may be the reason why “*Ca.* Nitrosacidococcus urinae” was selected instead of *Nitrosoglobus* sp. The DO affinity constant for the acid-tolerant AOB was estimated to be approximately k_S,DO_ = 0.8 mg L^-1^ (SI 25). The value is similar to the affinity constant of 1 mg L^−1^ reported for *Ca.* Nitrosoglobus sp. (Wang et al. 2021a) and within the range of 0.1 to 1.45 mg L^−1^ used for wastewater treatment models (Sin et al. 2008).

#### 3.5.3 Acid-sensitive AOB related to *Nitrosomonas halophila* outcompeted acid-tolerant AOB at pH 7 potentially due to iron limitation

**Fig. 7:**
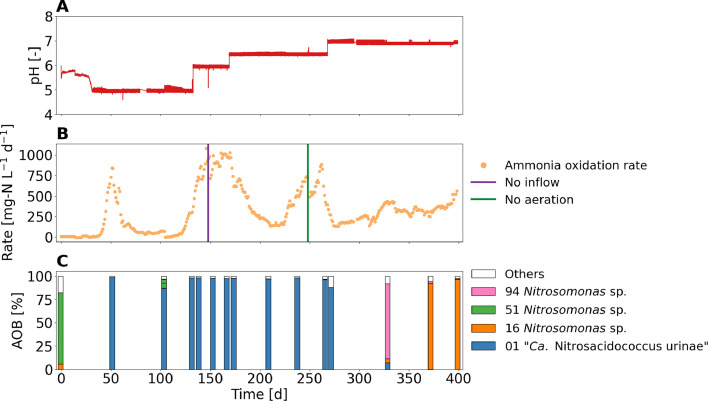
Performance of the urine nitrification reactor operated for 400 days at pH values between 5 to 7. (A) pH. (B) Ammonia oxidation rate, nitrite oxidation rate and operational disturbances. (C) Relative read abundance of AOB species (OTUs) compared to all recognised AOB species.

In the long-term pH experiment, high ammonia oxidation rates of up to 840 mg-N L^−1^ d^−1^, 1060 mg-N L^-1^ d^−1^ and 880 mg-N L^−1^ d^−1^ were observed for pH 5, pH 6 and pH 6.5, respectively (Fig. 7A and B). The maximum ammonia oxidation rate at pH 7 was 570 mg-N L^−1^ d^−1^. Disturbances in reactor operation were less problematic at higher pH than at pH 5. The ammonia oxidation rate recovered rapidly after a brief influent stop and a 16-hour interruption of aeration on day 148 and 248, respectively (Fig. 7B). As already shown in Fig. 5, anoxic phases are less of a problem at pH values higher than pH 5. “*Ca.* Nitrosacidococcus urinae” was the dominant AOB in a pH range from 5 to 6.5, but at pH 7, acid-sensitive AOB (OTU 16) related to the *Nitrosomonas halophila* lineage (see SI 26 for phylogenetic tree) took over (Fig. 7C). OTU 16 was the second most abundant AOB species in the inoculum after OTU 51, which was closely related to *Nitrosomonas europaea*. The switch from acid-tolerant to acid-sensitive AOB was also evident in the pH batch experiment, as the batches with activated sludge from day 360 onwards did not decrease below a pH of 5.4 (batch experiments in SI 27). No potential NOB were found from the activated sludge operated at pH values 6, 6.5, and 7 (see the relative abundance of *Nitrobacter* and *Xanthobacteraceae* linked OTUs in SI 28).

The maximum observed net growth rates at pH 5, 6 and 6.5 were around 0.6 d^−1^ (see SI 29), which corresponds to the value of the enrichment reactor (Section 3.2). Nevertheless, higher nitrification rates were obtained compared to the nitrification reactor due to higher influent concentrations. Since the SRT is equal to the HRT, and the flow rate depends on the ammonia oxidation rate, given the same growth rate, a lower maximum ammonia oxidation rate will be achieved with a lower TAN concentration in the feed. At pH 7 the maximum observed net growth rate decreased to 0.1 d^-1^ within less than a week and most likely only increased once acid-sensitive AOB took over. Nitrite oxidation rates almost completely stopped at pH 6 (NAR > 98 %) and above. Biological nitrite oxidation was most likely inhibited initially by the HNO_2_ concentration and at pH 7 possibly by the combined inhibitory effect of HNO_2_ and NH_3_ preventing the regrowth of NOB. The chemical nitrite oxidation rate decreased with increasing pH due to the lower HNO_2_ concentrations at pH 6 (3 mg-N L^−1^), pH 6.5 (1 mg-N L^−1^) compared to pH 5 (15 mg-N L^−1^) (see simulations in SI 13). HNO_2_ inhibition is also most likely the reason why the dominant AOB at pH values 6 and 6.5 is the acid-tolerant “*Ca.* Nitrosacidococcus urinae” I8 instead of acid-sensitive AOB linked to *Nitrosomonas* sp., which were found in partial urine nitrification at pH values between 5.8 and 7 (Fumasoli et al. 2016). At HNO_2_ concentrations of about 1 mg-N L^−1^, acid-sensitive AOB of the *N. europaea* cluster showed inhibition of 90% (Faust et al. 2022a). Zuo et al. (2022) successfully operated a partial urine nitritation system at pH 5.9 to 8, converting approximately 50% of TAN to nitrite, but they did not analyze the microbial community. It is possible that for at least part of the experimental period, the main AOB was also an acid-tolerant AOB. While partial nitritation of high-strength nitrogenous influent is usually operated at alkaline pH to ensure fast growth of AOB without NH_3_ substrate limitation (Jubany et al. 2009), acid-tolerant AOB such as “*Ca.* Nitrosacidococcus urinae” I8 allow high growth rates even at pH around 6 due to their high affinity for NH_3_ and their tolerance for HNO_2_. The results suggest that systems for partial nitritation of high-strength nitrogenous influents should be operated at pH 6 or above, where chemical and biological nitrite oxidation are low and process disturbances are less critical.

**Fig. 8:**
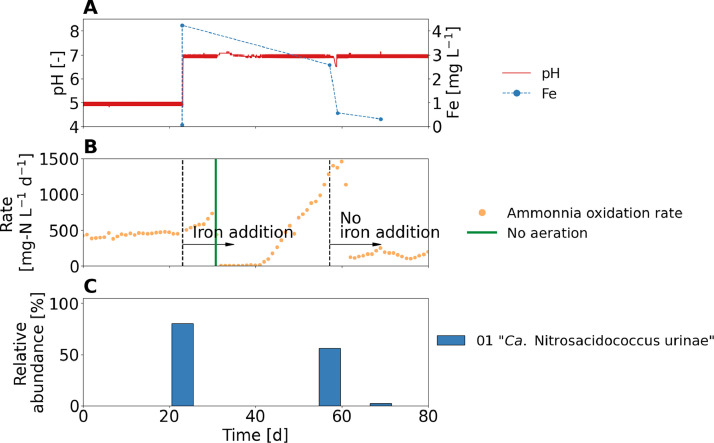
Performance of the urine nitrification reactor operated at pH 5 and 7 with iron addition. (A) pH and total iron (Fe) concentrations in the reactor. After 22 days, pH was increased and iron was added to the reactor and the influent. After 57 days, influent without iron addition was used. The concentrations of iron and other trace elements in the influent and in the reactor can be found in SI 32. (B) Ammonia oxidation rates and indication of one operational failure (vertical line) during which aeration was turned off for 72 h. (C) Relative read abundance of “Ca. Nitrosacidococcus urinae”. The relative abundance of acid-sensitive AOB was less than 0.01%.

The net growth rate of the acid-tolerant AOB decreased strongly at pH 7, which was not observed in the short-term experiments. Looking at the pH-dependent processes, iron stands out as it is less abundant at high pH due to the iron complexation equilibrium (see Fe^2+^ and Fe^3+^ speciation in SI 30), as high pH increases chemical iron oxidation and decreases the solubility of iron (Ferguson and Ingledew 2008). Iron it is an important element for nitrifiers because hydroxylamine oxidoreductase (HAO) contains iron-containing cytochromes (Liu et al. 2014). Another important element for nitrifiers is coppers as it is a component of the enzyme ammonia monooxygenase (AMO) (Musiani et al. 2020), but the speciation of copper only shows little pH dependence (see Cu^+^ and Cu^2+^ speciation in SI 30). Therefore, it was hypothesized that the decrease in activity might be related to iron limitation. When additional iron was dosed into the reactor and influent, a change in pH from 5 to 7 did not lead to a decrease of the ammonia oxidation rate or “*Ca.* Nitrosacidococcus urinae” abundance (Fig. 8). Despite a reactor disturbance without airflow, ammonia oxidation rates of up to 1500 mg-N L^−1^ d^−1^ were achieved, which was higher than at any other pH values. Shortly after changing the influent to urine without iron addition, the activity decreased again down to a growth rate of about 0.1 d^−-1^ (see SI 31) and “*Ca.* Nitrosacidococcus urinae” was washed out.

The enzymes found in “*Ca.* Nitrosacidococcus urinae” I8 associated with iron transport could be a possible explanation for the observed correlation between the growth of “*Ca.* Nitrosacidococcus urinae” and the iron availability. In general, there are two transport mechanism based on either siderophore system or direct iron transport (Vajrala et al. 2010). Siderophore systems allow microorganisms to scavenge iron from precipitates under iron-limited conditions by synthesizing iron-chelating compounds (Krewulak and Vogel 2008). While *Nitrosomonas europaea* species possess a siderophore system and direct iron transporters (Chain et al. 2003), the genome of “*Ca.* Nitrosacidococcus urinae” I8, just like that of “*Ca.* Nitrosacidococcus tergens” RJ19, lacks a siderophore system. Instead, the “*Ca.* Nitrosacidococcus*”* species contain only a high affinity iron ion uptake system that also contains a cytochrome motif (CxxCH). The acid-tolerant AOB “*Ca.* Nitrosoglobus terrae” also does not contain a siderophore system (Hayatsu et al. 2017), but in general, the iron transport mechanism of nitrifiers are not very well studied. Thus, even though there was a strong correlation between the availability of dissolved iron and “*Ca.* Nitrosacidococcus urinae” at neutral pH, further experiment, e.g. using transcriptomic, are needed to better understand the iron uptake mechanism and the related iron limitation of acid-tolerant AOB.

## 4 Conclusions

• A novel acid-tolerant AOB, “*Ca.* Nitrosacidococcus urinae” I8, enables fast ammonia oxidation at low pH of high-strength nitrogenous influents such as source-separated urine.
• Ammonia oxidation under acidic conditions and high nitrogen levels is highly sensitive to process disturbances, such as uncontrolled pH changes or interruption of aeration, so careful operation and process control are required. In addition, chemical nitrite oxidation causes high nitrogen losses, mostly in the form of harmful nitrogen oxide gases.
• Acid-tolerant AOB are scarcely present in urine nitrification reactors operated at pH values above 5.8, but they enrich in phases without pH control and are thus relevant for the process.
• At increasing pH, the activity of “*Ca.* Nitrosacidococcus urinae” decreases, which strongly correlates with the limited availability of iron at higher pH and is possibly related to the absence of a siderophore system.

## CRediT authorship contribution statement

**Valentin Faust:** Conceptualization, Methodology, Investigation, Data curation, Writing – original draft, Writing – review & editing, Visualization. **Theo A. van Alen:** Methodology, Investigation, Data curation, Writing – review & editing, Visualization. **Huub J.M. Op den Camp:** Conceptualization, Methodology, Writing – review & editing. **Siegfried E. Vlaeminck:** Conceptualization, Methodology, Writing – review & editing, Supervision. **Ramon Ganigué:** Conceptualization, Methodology, Writing – review & editing, Supervision. **Nico Boon:** Conceptualization, Methodology, Writing – review & editing, Supervision. **Kai M. Udert:** Conceptualization, Methodology, Writing – review & editing, Supervision, Project administration, Funding acquisition.

## Declaration of Competing Interest

The authors declare that they have no known competing financial interests or personal relationships that could have appeared to influence the work reported in this paper.

## Data Availability

Data will be made available on request.

## Appendix Supplementary materials

Image, application 1
