# Supplementary material for: Ammonia oxidation by novel “Candidatus Nitrosacidococcus urinae” is sensitive to process disturbances at low pH and to iron limitation at neutral pH
Source: Water Res X. 2022 Oct 4;17:100157. doi: 10.1016/j.wroa.2022.100157 (PMC9574496; doi:10.1016/j.wroa.2022.100157)
Supplement: Supplementary file 1 [file mmc1.pdf]

Ammonia oxidation by novel "*Candidatus* Nitrosacidococcus urinae" is sensitive to process disturbances at low pH and to iron limitation at neutral pH

SUPPLEMENTARY INFORMATION

Valentin Faust[a,b], Theo A. van Alen[c], Huub J.M. Op den Camp[c], Siegfried E. Vlaeminck[d,e], Ramon Ganigué[e,f], Nico Boon[e,f], Kai M. Udert[a,b]

[a] Eawag, Swiss Federal Institute of Aquatic Science and Technology, 8600 Dübendorf, Switzerland

[b] ETH Zürich, Institute of Environmental Engineering, 8093 Zürich, Switzerland

[c] Department of Microbiology, RIBES, Radboud University Nijmegen, 0268 Nijmegen, the Netherlands

[d] Research Group of Sustainable Energy, Air and Water Technology, Department of Bioscience Engineering, Faculty of Science, University of Antwerp, 2020 Antwerpen, Belgium

[e] Centre for Advanced Process Technology for Urban Resource recovery (CAPTURE), Frieda Saeysstraat 1, 9052 Gent, Belgium

[f] Center for Microbial Ecology and Technology (CMET), Faculty of Bioscience Engineering, Ghent University, 9000 Gent, Belgium

Emails of the authors: valentin.faust@eawag.ch, t.vanalen@science.ru.nl, h.opdencamp@science.ru.nl, siegfried.vlaeminck@uantwerpen.be, ramon.ganigue@ugent.be, nico.boon@ugent.be, kai.udert@eawag.ch

Corresponding author: Kai M. Udert, kai.udert@eawag.ch

Contents

1 Main reactor set-up 4

2 Respirometer set-up 5

3 Chemical nitrite oxidation model: main reactor 8

4 Acid base equilibrium for ammonium and ammonia 12

5 Acid base equilibrium for nitrous acid and nitrite 14

6 Incubation experiments 16

7 pH evolution 17

8 Reactor operation and performance 19

9 Operational disturbances 22

10 Nitrite accumulation ratio 23

11 Estimation of nitrogen losses 24

12 Biological and chemical nitrite oxidation rate 25

13 Chemical nitrite oxidation rate vs. HNO_2 and DO 26

14 Relative abundance of main AOB 27

15 Phylogenetic tree "*Ca.* Nitrosacidococcus urinae" 28

16 Phylogenetic tree and relative abundance of potential NOB 29

17 Microbial diversity 31

18 Particle size distribution 32

19 Net growth rate 33

20 Influence of anoxic conditions (no aeration) 34

21 Influence of anoxic conditions (N_2 stripping) 35

22 Batch activity tests without pH control 36

23 NO concentration main reactor 37

24 Salinity 38

25 Influence of dissolved oxygen substrate limitation 39

26 Phylogenetic tree of OTU 16, 51, and 94 40

27 pH drop experiments 41

28 NOB pH long-term experiment 42

29 Net growth rate long-term pH experiment 43

30 PHREEQC simulation of dissolved iron (Fe^{2+}, Fe^{3+}) and copper (Cu^{2+}, Cu^{+}) 44

31 Iron dosage long-term pH experiment – batch experiment 45

32 Iron dosage long-term pH experiment - dilution rate 46

33 Concentration of trace elements 47

34 References 48

1 Main reactor set-up

The main reactor for the enrichment of acid-tolerant AOB consisted of a continuous-flow stirred-tank reactor (CSTR) without sludge retention (hydraulic retention time = solid retention time) as shown in **Figure S1**. The reactor was fed from the middle with a peristaltic pump (PD-5001, Heidolph) using pumping tubes with a wall thickness of 1.6 mm and an inner diameter of 1.6 mm. For the effluent, the reactor had an overflow on the top. The reactor was equipped with an overhead stirrer (RZR 2020 Overhead Stirrer, Heidolph), a pressure gauge (Cerabar T PMC131, Endress+Hauser), a pH sensor (Orbisint CPS11D, Endress+Hauser), and a DO sensor (Oxymax COS61D, Endress+Hauser). The temperature was measured with the pH sensor (Orbisint CPS11D, Endress+Hauser) as well. The temperature was controlled via a water heat jacket (FN25, Julabo). For the main enrichment phase (700 days) only the lower temperature limit was controlled resulting in temperatures between 25-33°C. In the long-term pH experiment the lower and higher temperature limit was controlled resulting temperatures around 25°C. For aeration, pre-humidified air was used for the first 700 days to minimize the influence of evaporation.

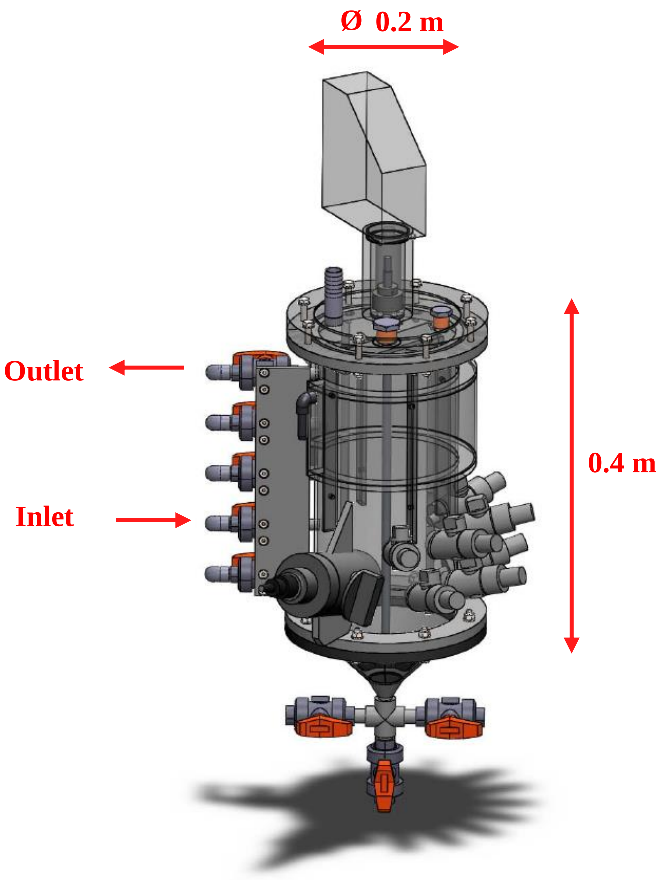

Figure S1: Dimension of the 12-L lab scale reactor used for the enrichment of acid-tolerant AOB and the long-term pH experiment (picture © Adriano Joss, Eawag).

2 Respirometer set-up

The respirometer consisted of a 2-L aeration chamber and a 0.7-L respiration chamber (**Figure S2**). While the aeration chamber was open to the atmosphere, the respiration chamber was completely closed. The two chambers were connected by a peristaltic pump (5IK40GN-CT, Watson Marlow) with a flow rate of 45.5 L h^{-1}. The pH in respirometer was controlled with 0.4 M NaOH and 0.4 M HCl using two peristaltic pumps (Reglo 897, Ismatec). Temperature was controlled at 25°C via a water heat jacket (FN25, Julabo). The aeration chamber was equipped with an overhead stirrer (RZR 2020 Overhead Stirrer, Heidolph), a pH sensor (Orbisint CPS11D, Endress+Hauser), and a DO sensor (Memosens COS81D, Endress+Hauser). The respiration chamber was equipped with a magnetic stirrer (Hei-Mix L, Heidolph), a DO sensor (Memosens COS81D, Endress+Hauser) and a pH sensor (Orbisint CPS11D, Endress+Hauser). For aeration, pre-humidified air was used to avoid evaporation.

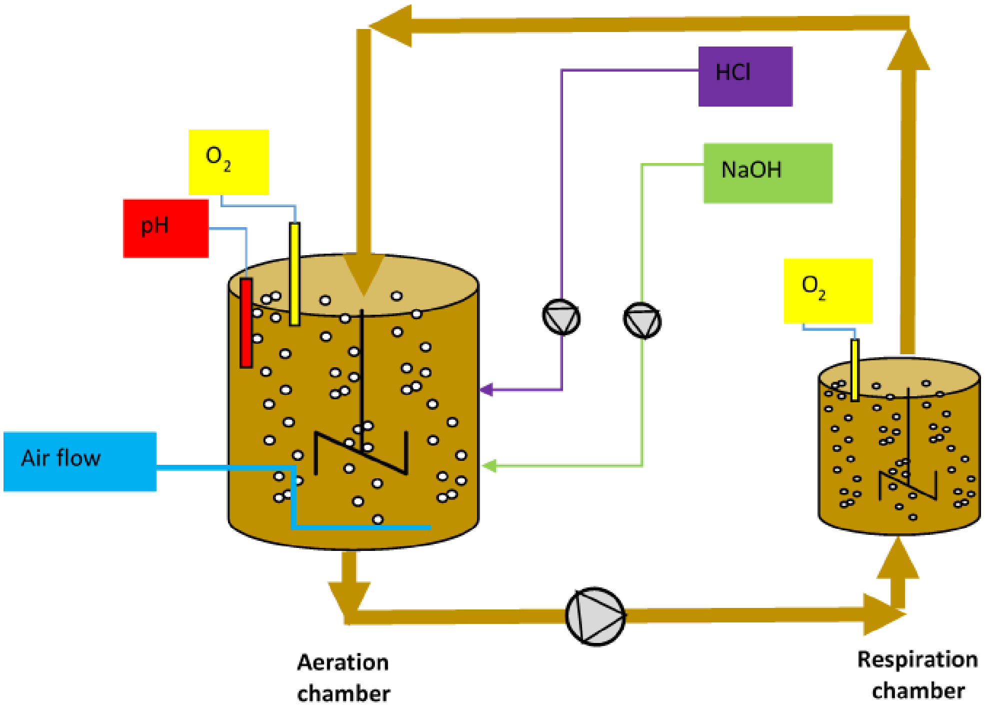

Figure S2: Two-chamber respirometer with a 2-L aeration chamber and a 0.7-L respiration chamber.

The respirometer was either operated as two chamber LSF (static gas, flowing liquid) respirometer or two chamber LSS (static gas, static liquid) respirometer. In the LSF respirometer, the aeration chamber was aerated to reach high DO concentration and the circulation pump continuously transported activated sludge to the respiration chamber. The oxygen uptake rate (OUR) [mg L^{-1} h^{-1}] was calculated using the DO mass balance over respiration chamber as shown in **Equation S1**,

$$\mathrm{OUR} = \frac{Q}{V_{resp}} * \left(DO_{aer} - DO_{resp}\right) - \frac{dDO_{resp}}{dt} \qquad (S1)$$

where Q [L h^{-1}] is the pump rate of the circulation pump, V_{resp} [L] the volume of the respiration chamber, DO_{aer} [mg L^{-1}] the DO in the aeration chamber assuming that it is the same concentration entering the respiration vessel, and DO_{resp} [mg L^{-1}] the DO in the respiration chamber assuming that it is the same concentration leaving the respiration vessel. In the LSS respirometer, the aeration chamber was aerated to reach high DO concentration and the circulation pump transported activated sludge with high DO concentrations to the respiration chamber using DO set-points between 4 to 6 mg L^{-1} and an on-off controller. When the upper DO set-point is reached, the circulation pump is switched off until the lower DO set-point is reached and the DO is switched off again. During the phase without pumping, the DO mass balance simplifies to **Equation S2**.

$$\mathrm{OUR} = -\frac{dDO_{resp}}{dt} \qquad (S2)$$

Due to the closed nature of the respiration chamber, no surface aeration or oxygen transfer coefficient has to be included. The nitrogenous oxygen uptake rate (NOUR) was determined by adding 10 mg/L N-allylthiourea (ATU) and substracting the remaning heterotrophic oxygen uptake rate, which was usually very small, from the OUR. The oxygen consumption due to nitrite oxidation was neglected as it consisted of chemical nitrite oxidation only and was generally low. The experiments are described in detail below.

Anoxic phases

To determine the effects of anoxic phases at different pH values, two experiments were conducted using the two chamber LSS respirometer. In the first experiment, 3 L of activated sludge from the main enrichment reactor operated at pH 5 was added to the respirometer and the pH was increased to 7 with NaOH. The respirometer was controlled at pH 7 for one hour. Subsequently, the aeration was turned off during 2.5 hours before re-aeration. After another one hour at pH 7, the pH was lowered to 5 with HCl. At pH 5, the reactor was operated for one hour before the aeration was stopped for another 2.5 hours. Last but not least, the respirometer was again operated at pH 5 for one hour. In the second experiment, 3 L of activated sludge from the main enrichment reactor operated at pH 5 was added to the respirometer. First, the reactor was operated at pH 5 for one hour. Then, the airflow was replaced by supplying N_2 gas instead during one hour. Afterwards, the aeration was reconnected and the reactor was operated at pH 5 for 2 hours.

pH effect, NH_3 inhibition, HNO_2 inhibition, and salinity

The short-term experiments on the influence of pH, NH_3, HNO_2, and salinity are summarised in **Table S1**. For all short-term experiments, 3 L of activated sludge from the main enrichment reactor operated at pH 5 was added to the respirometer. Once the oxygen uptake rate (OUR) was constant, the pH set-points were changed, $NaNO_2$ was added, or NaCl was added. Finally, ATU was added to determine the nitrogenous oxygen uptake rate (NOUR).

Table S1: Short-term experiments to evaluate the influence of pH, NH_3, HNO_2, and salinity. The effect of NH_3 inhibition and pH were studied together, and no TAN was added. Instead, the experiments were conducted during periods when the activated sludge had different TAN concentrations due to the different TAN concentration in the influent.

Target	Operation	TAN [mg-N L^{-1}]	TNN [mg-N L^{-1}]	Salinity [mS cm^{-1}]	pH [-]
pH/NH_3	LSS	450	390	10	5.0, 5.5, 6.0, 6.5, 7.0, 7.5, 8.0
pH/NH_3	LSF	670	620	14.5	5.0, 6.0, 7.0 ,8.0, 8.5
pH/NH_3	LSS	1235	980	22	5.0, 5.5, 6.0, 6.5, 7.0, 7.5, 8.0, 8.5
HNO_2	LSS	950	750, 1350, 1900, 2500	17, 20, 23, 26	5.0
Salinity	LSS	400	360	9, 12, 15, 21, 27, 32, 38, 48	5.0

DO substrate limitation

For a rough estimate of the oxygen affinity, 3 L of activated sludge was added to the LSS two chamber respirometer. The respirometer was aerated and continuously pumped to the respiration chamber until the DO in the respirometer chamber exceeded 5 mg L^{-1}. Subsequently, the pumping was stopped, and the DO decreased due to microbial activity. Based on the slope of the DO at different oxygen concentrations, the substrate affinity was estimated. The experiment was repeated three times with sludge from different time periods.

3 Chemical nitrite oxidation model: main reactor

Chemical nitrite oxidation was integrated in the wastewater treatment software SUMO19 developed by Dynamita (France) according to the processes and process rates in **Table S2** (Udert et al. 2005) in a Sumo2 model.

Table S2: Added processes and process rates in the Sumo2 model. All concentrations in [mol L^{-1}]. f_{mono} = activity coefficient for monovalent ions. All processes, process rates and kinetic constants according to (Udert et al. 2005). To describe the gas exchange of NO and NO_2, the simplified desorption equation for gases with low solubility (large Henry constant) was used, and for HNO_2 the simplified desorption equation for gases with high solubility (small Henry constant) was used (Crittenden et al. 2012).

Process	Process rate	Kinetic constant
**Nitrogen compounds equilibria**		
$2\ HNO_2 \rightarrow NO + NO_2 + H_2O$	$k_{NO,for} \times [HNO_2]^2$	$k_{NO,for} = 1.6 \times 10^6$ L mol^{-1} d^{-1}
$NO + NO_2 + H_2O \rightarrow 2\ HNO_2$	$k_{NO,back} \times [NO] \times [NO_2]$	$k_{NO,back} = 1.4 \times 10^{13}$ L mol^{-1} d^{-1}
$2\ NO_2 + H_2O \rightarrow HNO_2 + NO_3^- + H^+$	$k_{NO3,for} \times [NO_2]^2$	$k_{NO3,for} = 6.9 \times 10^{12}$ L mol^{-1} d^{-1}
$HNO_2 + NO_3^- + H^+ \rightarrow 2\ NO_2 + H_2O$	$k_{NO3,back} \times [HNO_2] \times f_{mono} \times [\ NO_3^-] \times f_{mono} \times [H^+]$	$k_{NO3,back} = 730$ L^2 mol^{-2} d^{-1}
$NO + NO_2 \rightarrow N_2O_3$	$k_{N2O3,for} \times [NO_2] \times [NO]$	$k_{N2O3,for} = 9.5 \times 10^{13}$ L mol^{-1} d^{-1}
$N_2O_3 \rightarrow NO + NO_2$	$k_{N2O3,back} \times [N_2O_3]$	$k_{N2O3,back} = 3.0 \times 10^9$ L mol^{-1} d^{-1}
**Chemical nitrogen conversion**		
$N_2O_3 + NH_3 \rightarrow N_2 + HNO_2 + H_2O$	$k_{NH3,nitro} \times [NH_3] \times [N_2O_3]$	$k_{NH3,nitro} = 7.7 \times 10^{10}$ L mol^{-1} d^{-1}
$2\ NO + O_2 \rightarrow 2\ NO_2$	$k_{NO,ox} \times [NO]^2 \times [O_2]$	$k_{NO,ox} = 1.8 \times 10^{11}$ L mol^{-1} d^{-1}
**Gas exchange**		
NO (aq) → NO (g)	$k_La_{NO} \times [NO]$	$k_La_{NO} = 164\ d^{-1}$, See below
NO_2 (aq) → NO_2 (g)	$k_La_{NO2} \times [NO_2]$	$k_La_{NO} = 104\ d^{-1}$, See below
HNO_2 (aq) → HNO_2 (g)	$Q_{air} * H_{HNO2} \times [HNO_2]$	$H_{HNO2} = 8.3*10^{-4}\ mol_g\ mol_{aq}^{-1}$

The average airflow (Q_{air}) during the experiment was about 2.5 L min^{-1}. The k_La for oxygen was estimated using **Equation S3**, where $C_{S,O2}$ is the saturation concentration, $C_{L,O2,t}$ the oxygen concentration at time t, $C_{L,O2,t0}$ the oxygen concentration at the beginning (t_0) and k_La_{O2} the volumetric mass transfer coefficient for oxygen (**Figure S3**).

$$\ln\left|\frac{C_{S,O2} - C_{L,O2,t}}{C_{S,O2} - C_{L,O2,t0}}\right| = -k_La_{O2} * (t - t_0) \qquad (S3)$$

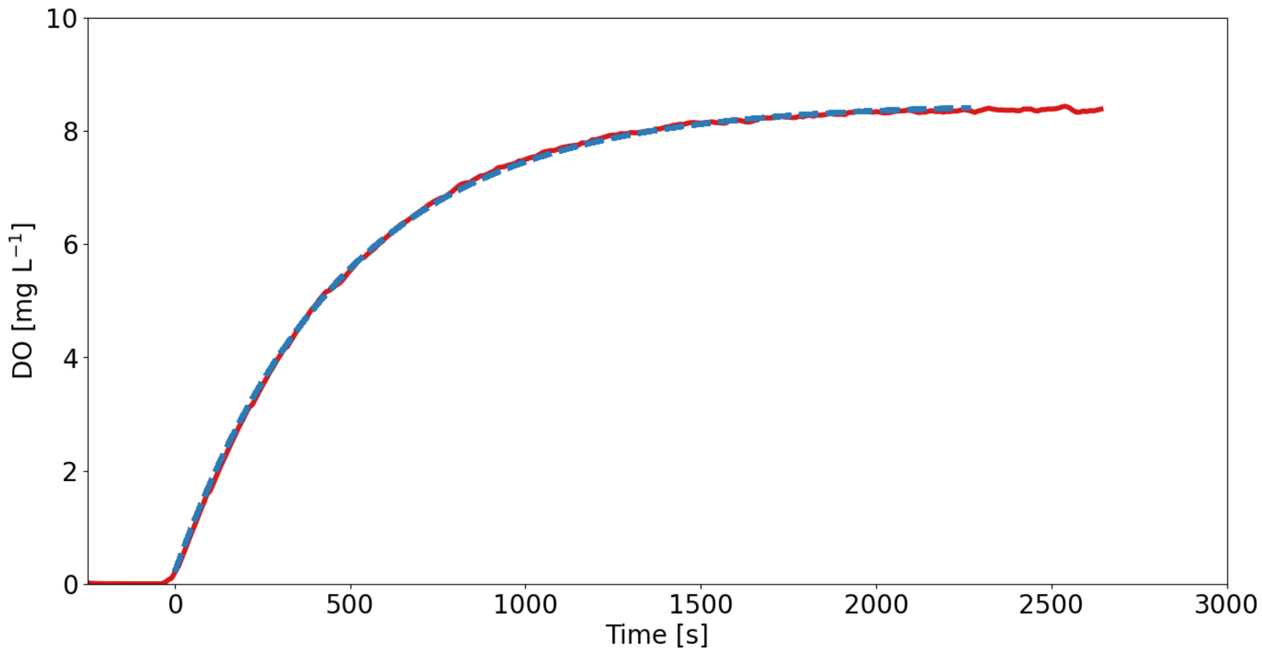

Figure S3: The k_La_{O2} of the 12-L reactor was estimated to be 180 d^{-1} at a temperature of 25°C and an airflow rate of 2.5 L min^{-1}. For the experiment nitrified urine with pH 6.3 was used and ATU was added to inhibit the nitrification.

Using the relationship in **Equation S4** and **Equation S5** (Crittenden et al. 2012), the k_La_{NO} and k_La_{NO2} were calculated,

$$\frac{k_La_{NO}}{k_La_{O2}} = \frac{D_{NO}}{D_{O2}} \qquad (S4)$$

$$\frac{k_La_{NO2}}{k_La_{O2}} = \frac{D_{NO2}}{D_{O2}} \qquad (S5)$$

with diffusion coefficients of D_{NO} = 1.9 × 10^{-4} m^2 d^{-1} (Lide 2009), D_{NO2} = 1.2 × 10^{-4} m^2 d^{-1} (Zacharia and Deen 2005), and D_{O2} = 2.1 × 10^{-4} m^2 d^{-1} (SUMO 2019).

The modelled system for the main reactor is shown in **Figure S4** and **Table S3** and consisted of an aerated CSTR (Nitritation) with additional pH control (base and acid controllers). The pH control was added to ensure that the pH corresponds with the measured one. The amount of base and acid added in the model affected the ionic strength only slightly.

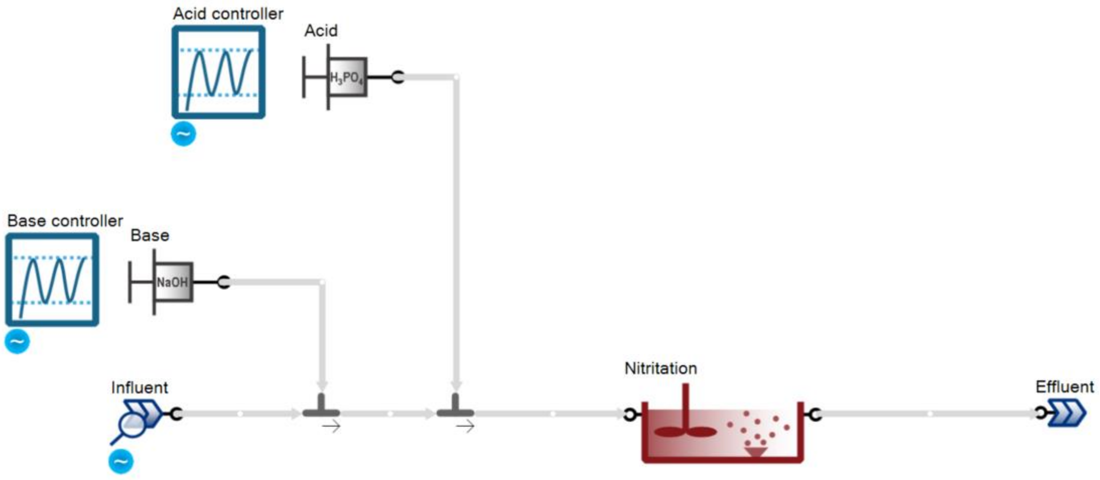

Figure S4: Sumo model process configuration for the main reactor. The nitrification was integrated as CSTR. The pH was kept at 5 with base (NaOH) and acid (H_3PO_4) controllers.

Table S3: Modified model input. The volume corresponds to the working volume during reactor operation.

**Variable**	**Value**
Volume	11.4 L
Tank depth	0.4 m
Air flow	2.5 L min^{-1}
Diffuser height from floor	0.05 m
Field air temperature	25°C
Influent temperature	25°C

The measured daily flow rates were used for the model input (**Figure S5A**). The pH in the model was kept at pH 5.5 for 29 days, and then at pH 5 except for the periods where operational failures were mimicked leading either to a pH drop or an uncontrolled pH increase (**Figure S5B**). It was not possible to model the acid-tolerant AOB directly due to the unexplainable fluctuations in activity. Therefore, the influent directly consistent of partially oxidized urine to circumvent the biological process. Based on the data of the enrichment reactor at pH 5, it was calculate that 49.7% of the TAN in the influent would be converted to nitrite by AOB (**Figure S5C**). The model calculates the ratio of TNN and nitrate due to chemical nitrite oxidation, which mainly depends on the residence time in the CSTR, the pH and the TNN concentration in the influent. Urine has a high ionic strength, which influences the chemical equilibria and reactions. To account for this, phosphate, sulfate, chloride, sodium and chloride were added in the simulated influent taking the values from (Fumasoli

et al. 2017) and scale them according to the TAN concentration in the influent. Airflow was at 2.5 L d^{-1}.

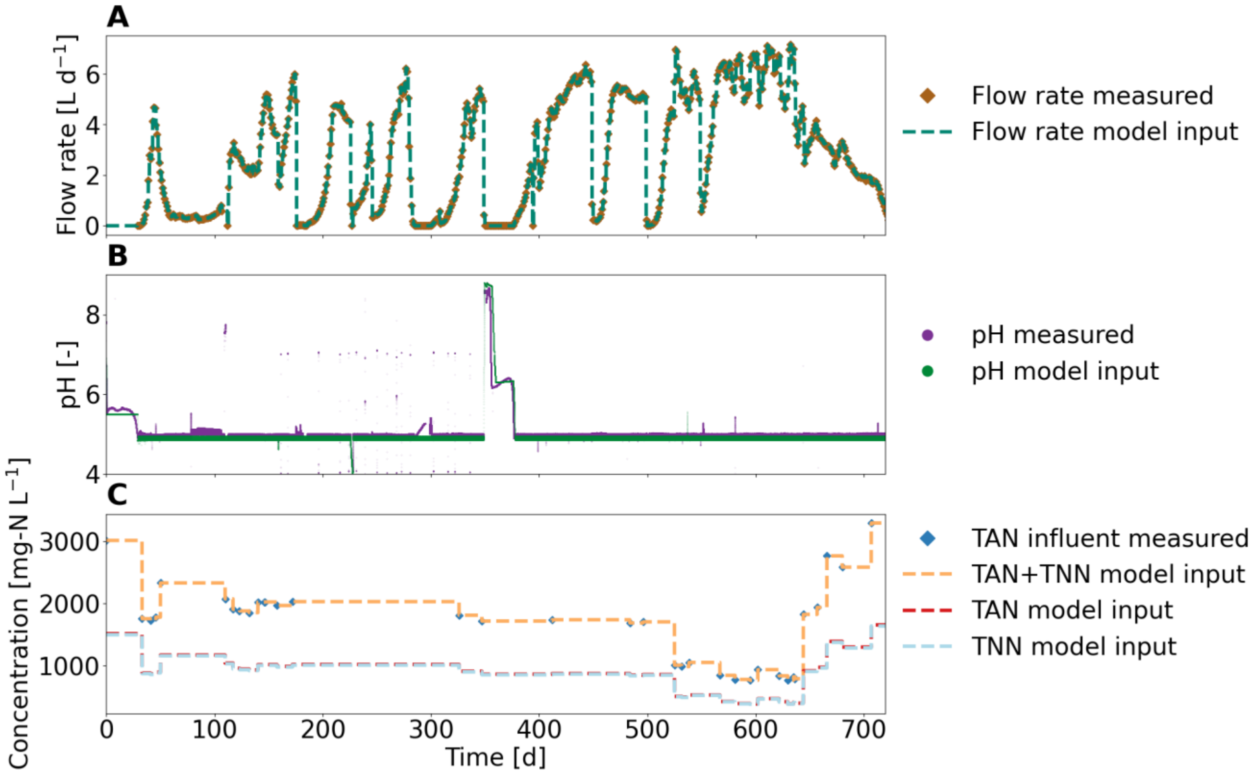

Figure S5: Dynamic input of the SUMO model and corresponding measured value. (A) Flow rate, (B) pH, and (C) inflow concentrations of the nitrogen species.

4 Acid base equilibrium for ammonium and ammonia

The acid-base equilibrium for ammonium and ammonia is shown in **Equation S6**.

$$NH_4^+ \leftrightarrow NH_3 + H^+ \qquad (S6)$$

Equilibrium concentrations were calculated according to **Equations S7**, **S8** (Crittenden et al. 2012), **S9** (Anthonisen et al. 1976), **S10** (Lewis and Randall 1921), and **S11** (Davies 1967) following the description in (Crittenden et al. 2012).

$$TAN = NH_3 + NH_4^+ \qquad (S7)$$

$$K_a(T) = \frac{NH_3 \times H^+ \times f_{mono}}{NH_4^+ \times f_{mono}} = \frac{NH_3 \times 10^{-pH}}{NH_4^+ \times f_{mono}} \qquad (S8)$$

$$K_a(T) = e^{\frac{-6344}{T}} \qquad (S9)$$

$$I = \frac{1}{2} \times \sum_i C_i \times Z_i^2 \qquad (S10)$$

$$\log_{10} f_{mono} = -A * \left(\frac{I^{0.5}}{1 + I^{0.5}} - 0.3 * I \right) \qquad (S11)$$

All variables are shown in **Table S4**.

Table S4: All variables used in the equations 6 to 11.

Variable	Name	Unit
TAN	Total ammonia nitrogen	[mol L^{-1}]
NH_3	Ammonia concentration	[mol L^{-1}]
NH_4^+	Ammonium concentration	[mol L^{-1}]
H^+	Proton concentration	[mol L^{-1}]
K_a	Dissociation constant, pK_a=9.25 @25°C	[mol L^{-1}]
f_{mono}	Activity coefficient for monovalent ions	[-]
T	Absolute temperature, 298 K @25°C	[K]
I	Ionic strength	[mol L^{-1}]
C_i	Concentration of ionic specie i	[mol L^{-1}]
Z_i	Charge of ionic specie i	[-]
A	0.51 at 25°C (Stumm and Morgan 1996)	[-]

For the calculation of the ionic strength, the main ionic species in nitrified urine were considered: K^+, Cl^-, Na^+, NH_4^+, NO_3^-, NO_2^-, $H_2PO_4^-$, and SO_4^{2-}. The ionic strength calculated for this publication were between 0.05 M to 0.2 M. For ionic strength below 0.1 M, the Davies equations (**Equation S11**) typically is in error by 1.5% and for ionic strength between 0.1 M to 0.5 M an error of 5% to 10% can be expected (Levine 1988).

5 Acid base equilibrium for nitrous acid and nitrite

The acid-base equilibrium for nitrous acid and ammonia is shown in **Equation S12**.

$$HNO_2 \leftrightarrow NO_2^- + H^+ \qquad (S12)$$

Equilibrium concentrations were calculated according to **Equations S13**, **S14** (Crittenden et al. 2012), **S15** (Anthonisen et al. 1976), **S16** (Lewis and Randall 1921), and **S17** (Davies 1967) following the description in (Crittenden et al. 2012).

$$TNN = HNO_2 + NO_2^- \qquad (S13)$$

$$K_a(T) = \frac{NO_2^- \times f_{mono} \times H^+ \times f_{mono}}{HNO_2} = \frac{NO_2^- \times f_{mono} \times 10^{-pH}}{HNO_2} \qquad (S14)$$

$$K_a(T) = e^{\frac{-2300}{T}} \qquad (S15)$$

$$I = \frac{1}{2} \times \sum_i C_i \times Z_i^2 \qquad (S16)$$

$$\log_{10} f_{mono} = -A * \left(\frac{I^{0.5}}{1 + I^{0.5}} - 0.3 * I\right) \qquad (S17)$$

All variables are shown in **Table S5**.

Table S5: All variables used in equations 12 to 17.

Variable	Name	Unit
TNN	Total nitrite nitrogen	[mol L^{-1}]
HNO_2	Nitrous acid concentration	[mol L^{-1}]
NO_2^-	Nitrite concentration	[mol L^{-1}]
H^+	Proton concentration	[mol L^{-1}]
K_a	Dissociation constant, pK_a=3.35 @25°C	[mol L^{-1}]
f_{mono}	Activity coefficient for monovalent ions	[-]
T	Absolute temperature, 298 K @25°C	[K]
I	Ionic strength	[mol L^{-1}]
C_i	Concentration of ionic specie i	[mol L^{-1}]
Z_i	Charge of ionic specie i	[-]
A	0.51 at 25°C (Stumm and Morgan 1996)	[-]

For the calculation of the ionic strength, the main ionic species in nitrified urine were considered: K^+, Cl^-, Na^+, NH_4^+, NO_3^-, NO_2^-, $H_2PO_4^-$, and SO_4^{2-}. The ionic strength calculated for this publication were between 0.05 M to 0.2 M. For ionic strength below 0.1 M, the Davies equations (**Equation S17**) typically is in error by 1.5% and for ionic strength between 0.1 M to 0.5 M an error of 5% to 10% can be expected (Levine 1988).

6 Incubation experiments

Several variables such as the operating pH before stopping the influent, the VSS concentration in the inoculum, or the average temperature of the incubation batch were plotted against the time required for the pH to drop below a value of 5, but no correlation was found (**Figure S6**). Therefore, the different duration of growth cannot be explained by the available data.

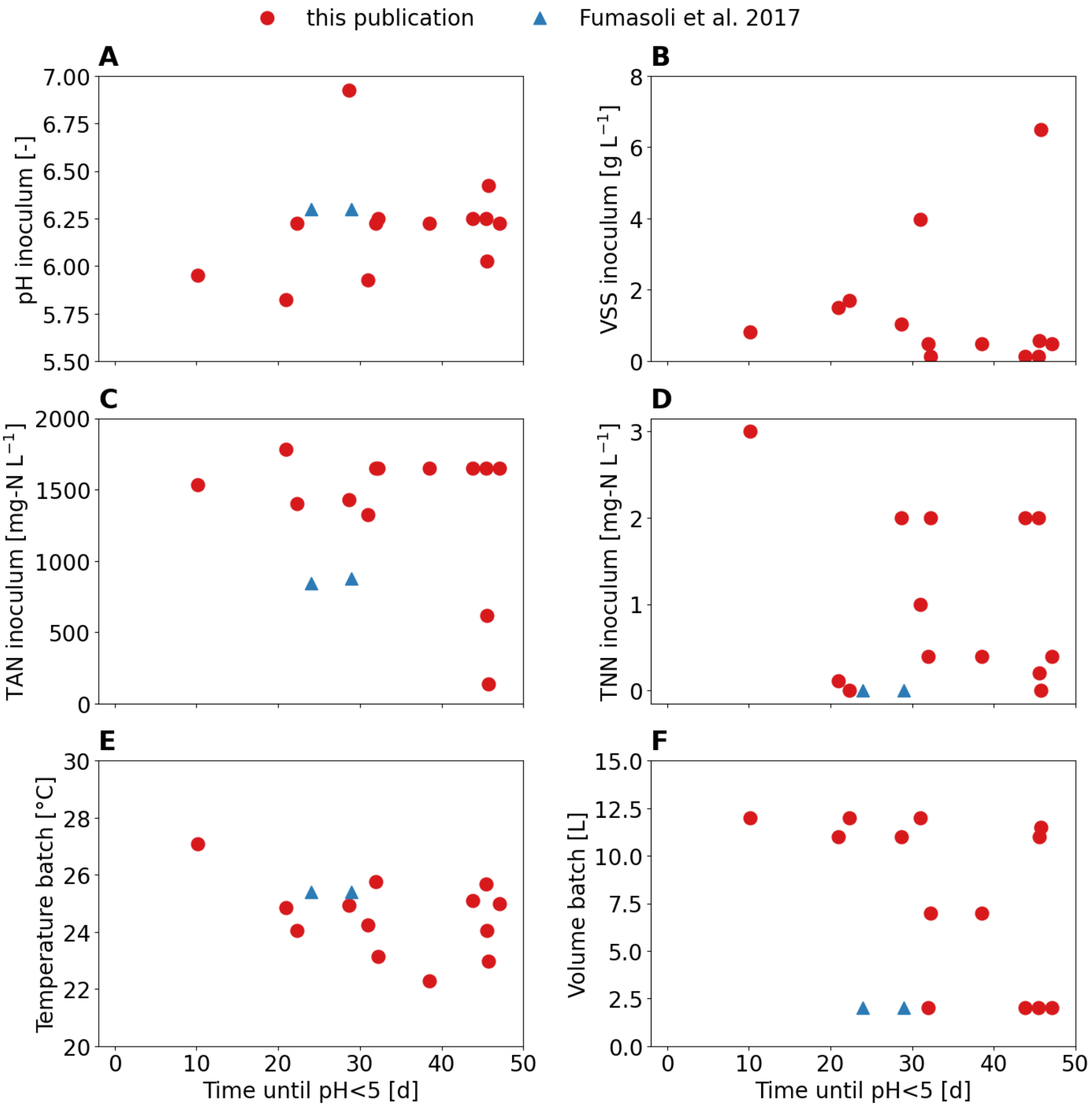

Figure S6: Scatterplot of various process variables against the time it took for the pH to fall below 5.0 indicating the growth of acid-tolerant AOB. Data from Fumasoli et al. (2017) were added to the data set. Variations in concentrations of other anions and cations such as phosphate or chloride were proportional to variations in TAN.

7 pH evolution

Table S6 lists the details of all inocula, and **Figure S7** shows the pH timelines of all incubation experiments.

Table S6: Origin and properties of all inocula used for the incubation batch experiments. * FC: 120-L urine nitrification reactor at Forum Chriesbach (Eawag, Switzerland), NEST: 160-L urine nitrification reactor at NEST (EMPA, Switzerland), Exp U: 12-L urine nitrification reactor from various experiments that were running for different purpose in the experimental hall (Eawag, Switzerland). Exp C: 12-L cow urine nitrification reactor from an experiment in the experimental hall (Eawag, Switzerland). + In all reactors, the pH was controlled with the influent using an on-off controller. The pH values give the upper and lower threshold.

ID	Start date	Inoculum origin*	pH [-]+	VSS [mg-N L^{-1}]	TAN [mg-N L^{-1}]	TNN [mg-N L^{-1}]	Temperature [°C]	Volume [L]
01	2018-09	FC	6.9 - 6.95	1040	1430	2	24.9	11
02	2019-08	NEST	6 - 6.05	570	620	0.2	24.1	11
03	2019-11	Exp U	5.8 - 5.85	1500	1780	0.11	24.8	11
04	2020-03	Exp U	6.2 - 6.25	490	1650	0.4	25.0	2
05	2020-03	Exp U	6.2 - 6.25	490	1650	0.4	25.8	2
06	2020-03	Exp U	6.2 - 6.25	490	1650	0.4	22.3	7
07	2020-03	Exp U	6 - 6.5	140	1650	2	25.1	2
08	2020-03	Exp U	6 - 6.5	140	1650	2	25.7	2
09	2020-03	Exp U	6 - 6.5	140	1650	2	23.2	7
10	2020-12	Exp C	6.4 - 6.45	6500	135	0	23.0	11
11	2021-10	FC	5.9 - 5.95	3980	1323	1	24.2	11
12	2022-01	NEST	5.9 - 6	809	1532	3	27.1	11
13	2021-12	NEST	6.2 - 6.25	1698	1404	0	24.1	11

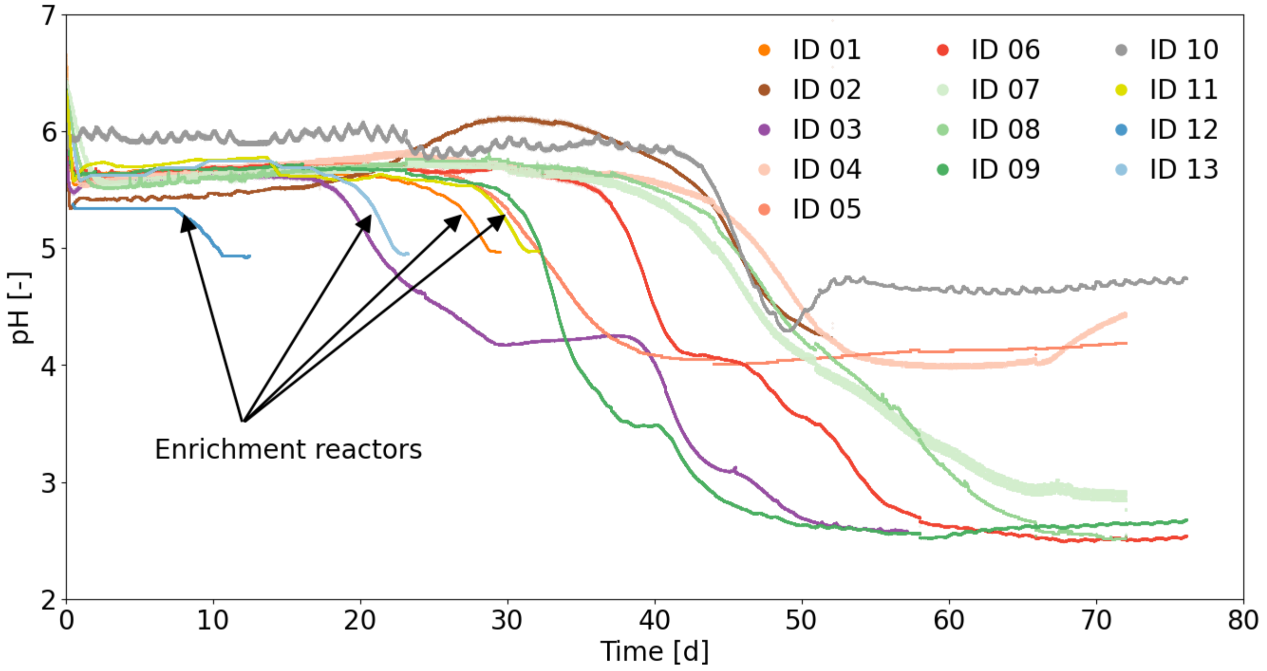

Figure S7: pH evaluation of all 13 incubation experiments. When the pH was not controlled at 5, the pH further decreased to values as low as 2.5, which was also observed by Fumasoli et al. (2017).

8 Reactor operation and performance

A statistical overview of all measured variables in the reactor and in the influent is shown in **Table S7, Table S8**, and Figure S8. In addition, the calculated HNO_2 and NH_3 (**Figure S9**) concentrations and the VSS concentration (**Figure S10**) in the reactor are shown.

Table S7: Reactor conditions and performance during the measurement campaign. The first 50 days were not taken into account as they were considered as start-up period.

		Mean	Stdv.	Min	Max
TAN	[mg-N L^{-1}]	840	170	380	1310
TNN	[mg-N L^{-1}]	630	160	630	930
Nitrate	[mg-N L^{-1}]	200	100	40	550
TSS	[mg L^{-1}]	220	110	40	570
VSS	[mg L^{-1}]	150	80	40	320
pH	[-]	5.0	0.2	3.9	8.5
DO	[mg L^{-1}]	5.3	1.0	0.0	8.4
Temperature	[°C]	24.7	3.6	20.4	33.4
Flow rate	[L d^{-1}]	2.9	2.2	0	7.2
Ammonia oxidation rate	[mg-N L^{-1} d^{-1}]	200	150	0	510
Nitrite oxidation rate	[mg-N L^{-1} d^{-1}]	40	35	0	290
Specific ammonia oxidation rate	[g-N g-VSS^{-1} d^{-1}]	2	2	0	10

Table S8: Concentrations, pH, and conductivity in the influent. The COD pre-treatment step reduced the influent COD by about 70 to 80%.

		Mean	Stdv.	Min	Max
TAN = NH_4^+-N + NH_3-N	[mg-N L^{-1}]	1880	765	765	3300
COD	[mg L^{-1}]	480	140	270	790
pH	[-]	8.7	0.3	8.3	9.4
Conductivity	[mS cm^{-1}]	16.1	4.6	9.5	26

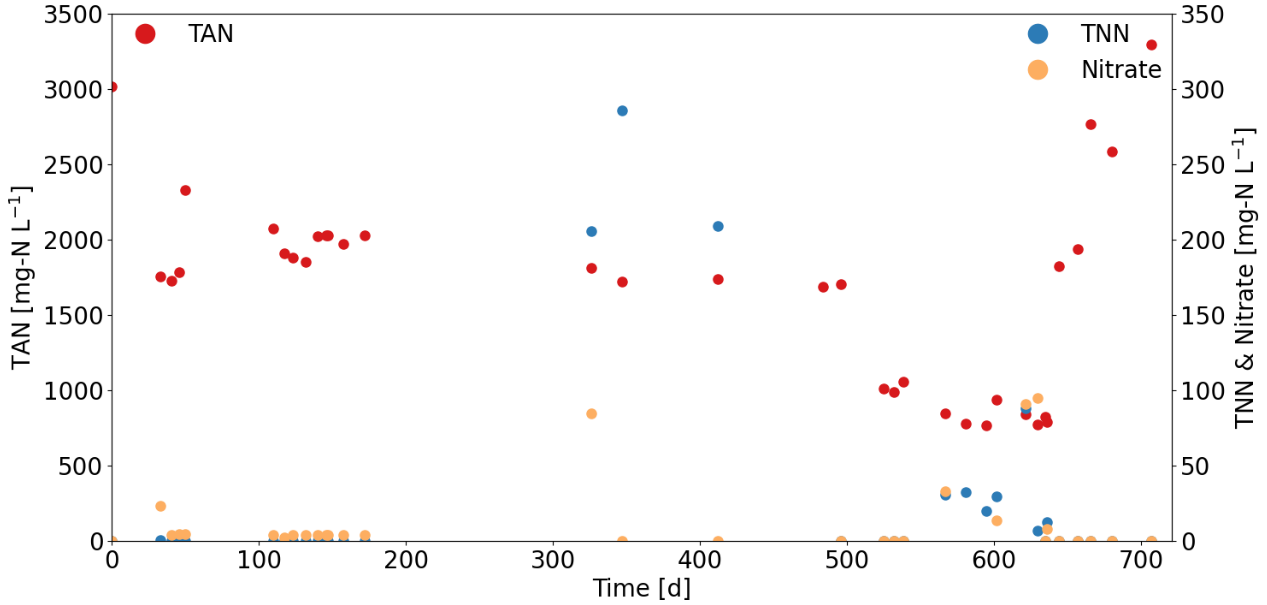

Figure S8: Concentrations of the main nitrogen species in the influent. TAN = NH_4^+-N + NH_3-N and TNN = NO_2^--N + HNO_2-N. No relevant nitrification occurred in the MABR itself, but some nitrification was observed when the MABR effluent was stored for an extended period of time before being used as influent for the enrichment reactor, e.g., after 350 days.

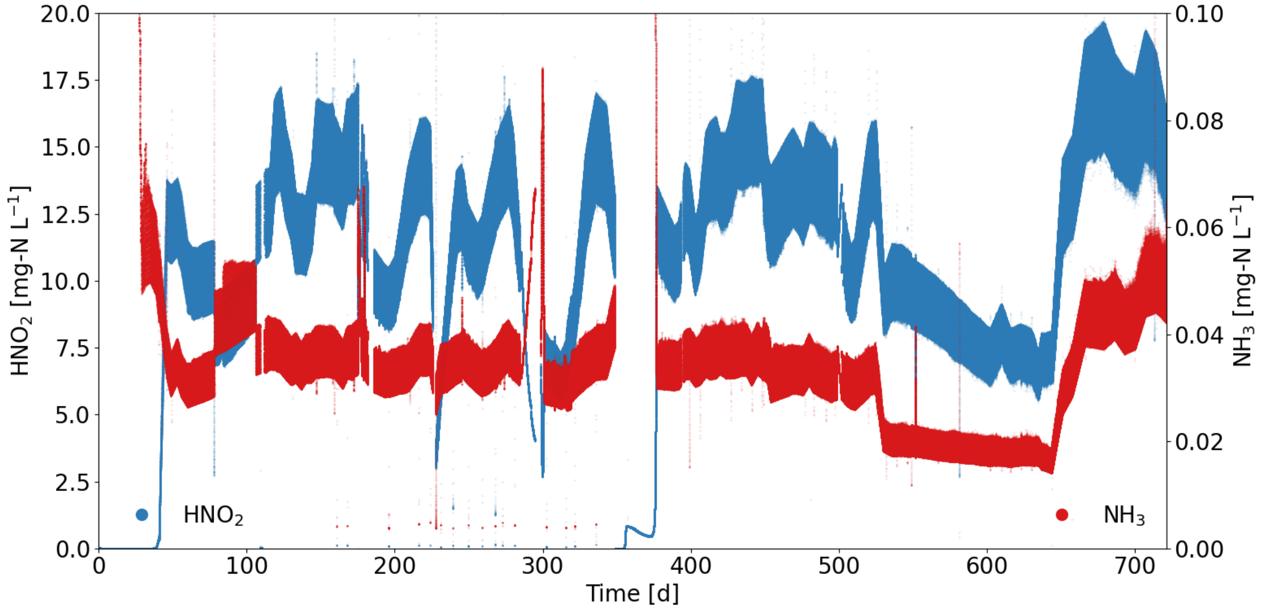

Figure S9: Calculated HNO_2 and NH_3 concentrations in the main enrichment reactor. The concentrations were calculated based on the measured pH and the interpolated TNN and TAN concentrations.

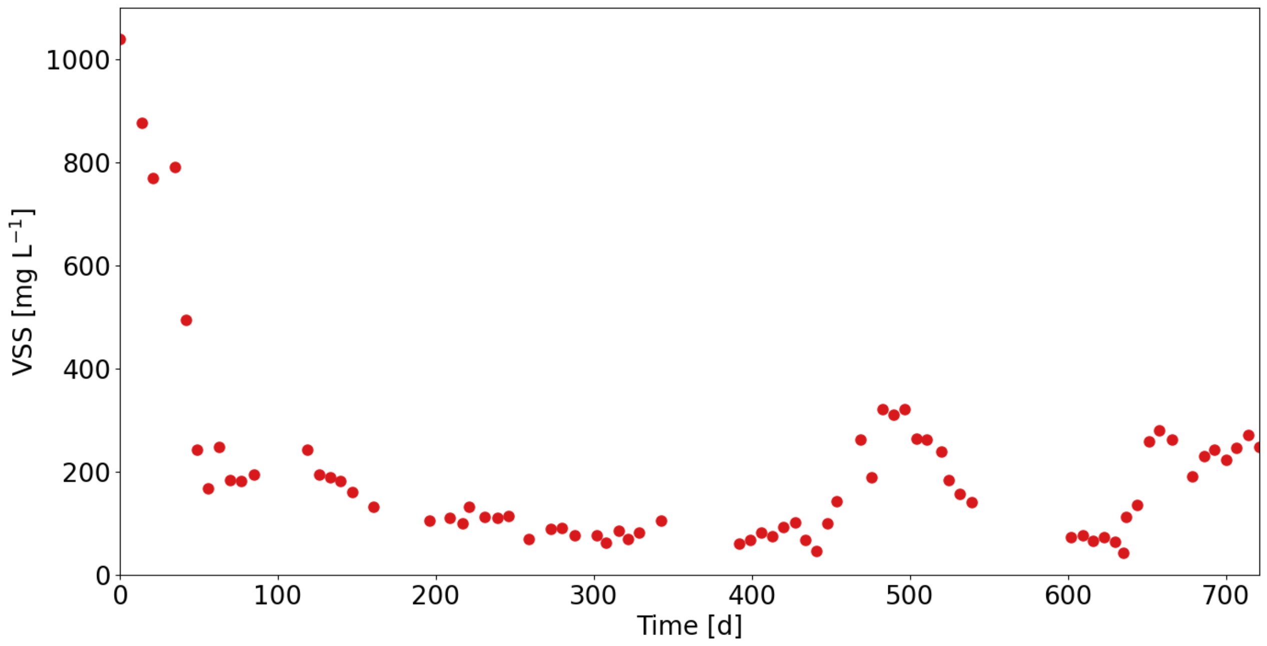

Figure S10: VSS concentrations decreased from 1000 mg-VSS L^{-1} to 200 mg VSS L^{-1} within the first 50 days. This was most likely related to the COD pretreatment step, which was not used to feed the nitrification reactor of the inoculum, resulting in a decrease in heterotrophic biomass.

9 Operational disturbances

Operational disturbances that occurred in the main enrichment reactor are listed in **Table S9**.

Table S9: Operational disturbances of the main enrichment reactor.

Time [d]	Duration	Operational disturbances	Effect
159	9 h	pH control stopped and aeration continued	pH decreased to 4.5
175	15 h	Aeration and pH control stopped	DO decreased to about 0 mg L^{-1}
226	52 h	pH control stopped and aeration continued	pH decreased to 4.0
245	3 h	Aeration and pH control stopped	DO decreased to about 0 mg L^{-1}
278	72 h	Very high outdoor temperatures	Temperatures increased to 33°C
348	12	Uncontrolled continuous aeration and pumping of 12 L	pH increased to 8.5
449	5 h	Aeration and pH control stopped	DO decreased to about 0 mg L^{-1}
498	12 h	Aeration and pH control stopped	DO decreased to about 0 mg L^{-1}
549	6 h	Aeration and pH control stopped	DO decreased to about 0 mg L^{-1}

10 Nitrite accumulation ratio

The nitrite accumulation ratio (NAR), i.e., the amount of nitrite that was not converted to nitrate, averaged 77% ± 12%, excluding the start-up phase (**Figure S11**). In general, a lower flow rate and a lower pH resulted in lower NAR.

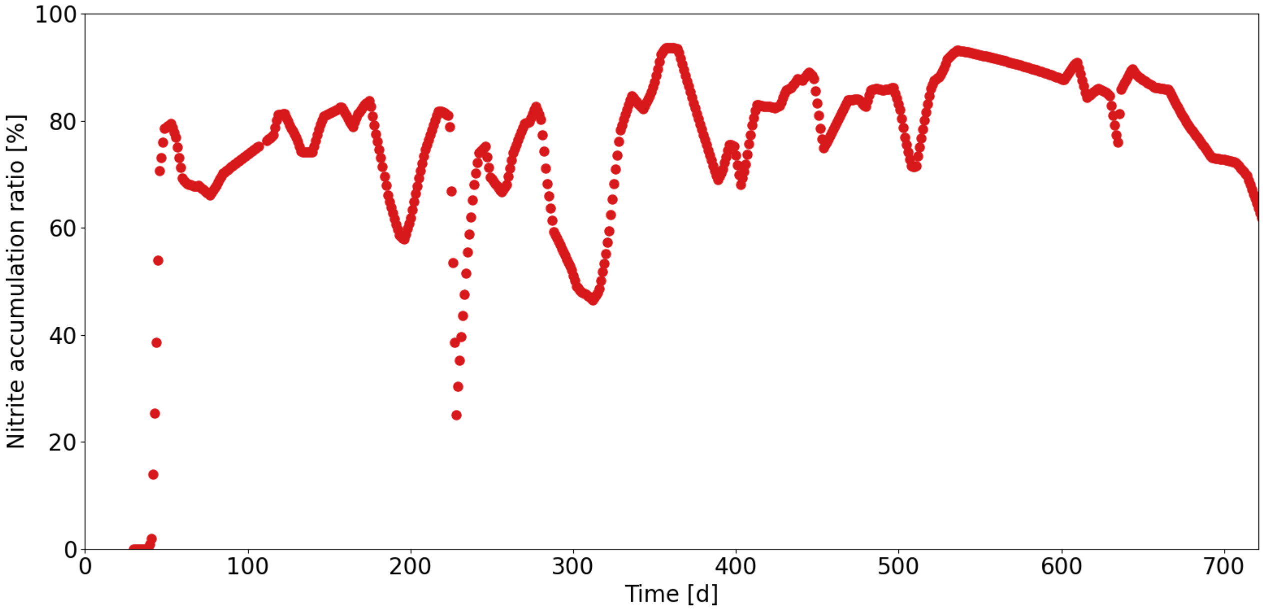

Figure S11: Nitrite accumulation ratio in the main enrichment reactor.

11 Estimation of nitrogen losses

Nitrogen losses were estimated by calculating the difference between the sum of TAN, TNN and nitrate nitrogen in the influent and in the effluent, and the concentration gradient in the reactor (**Figure S12**). Evaporation was assumed to be negligible because humidified air was used for aeration. Nitrogen incorporated into the biomass was also neglected. The first 50 days were not included because they were considered as start-up period. Nitrogen losses were approximately 11% of the nitrogen in the influent.

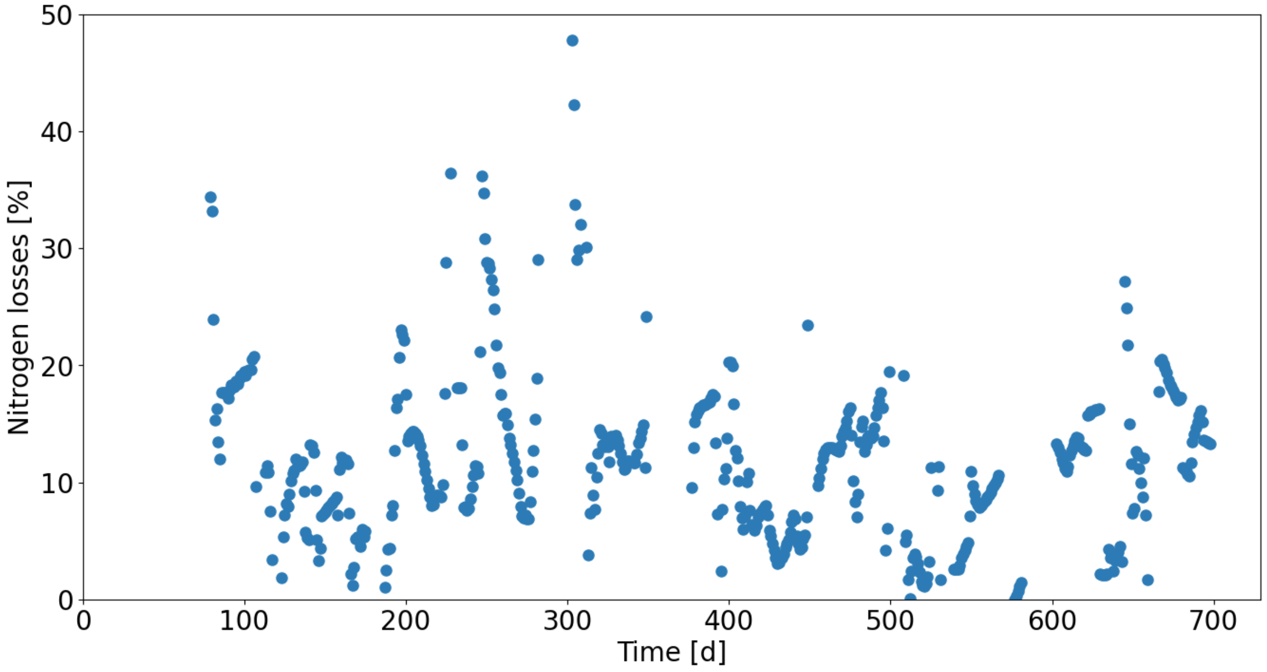

Figure S12: Nitrogen losses in the main enrichment reactor.

12 Biological and chemical nitrite oxidation rate

Only the first peak of nitrite oxidation around day 40 was not well captured by chemical nitrite oxidation (**Figure S13**). Most likely, the first peak was caused by biological nitrite oxidation, but once the HNO_2 concentration exceeded 0.5 mg-N L^{-1} around day 42, the rates decreased and chemical nitrite oxidation became dominant.

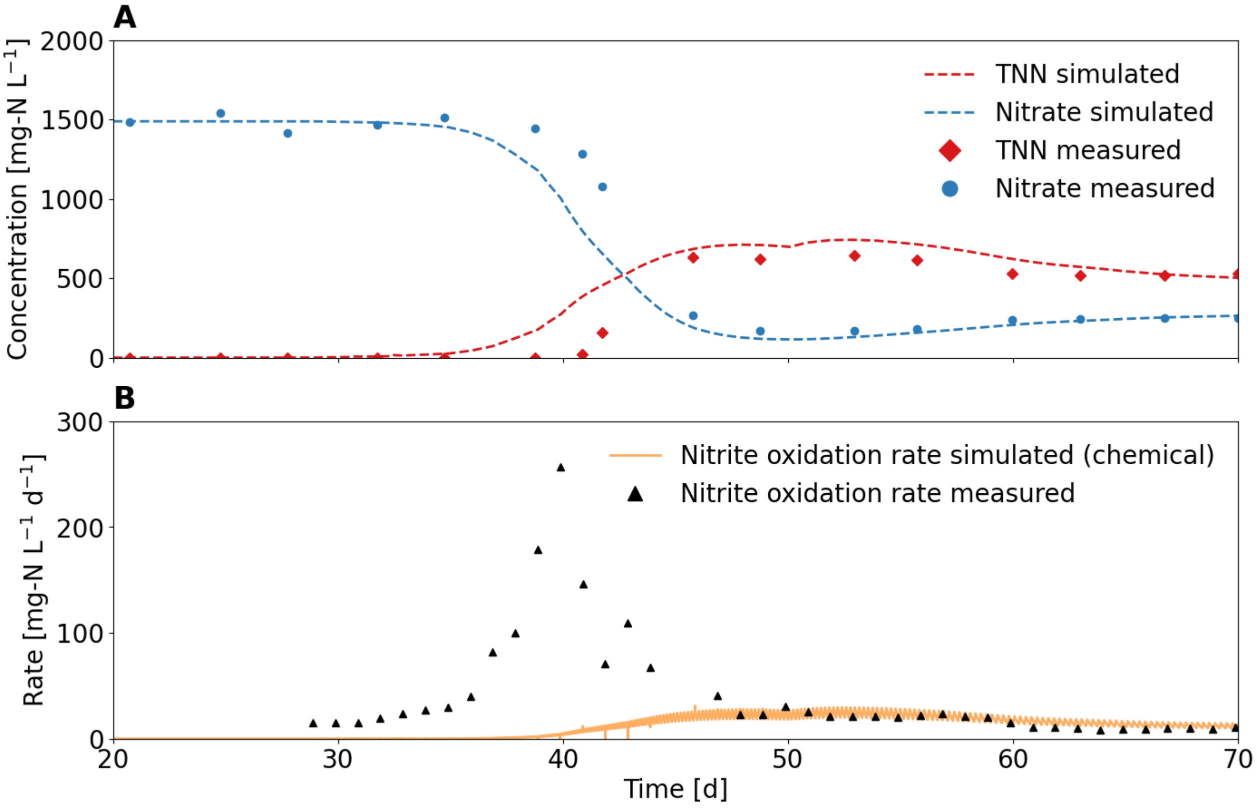

Figure S13: Simulation of chemical nitrite oxidation for the first peak of observed nitrite oxidation. (A) Measured and simulated TNN (= NO_2^--N + HNO_2-N) and nitrate concentrations in the reactor. (B) Measured nitrite oxidation rate and simulated chemical nitrite oxidation rate.

13 Chemical nitrite oxidation rate vs. HNO_2 and DO

According to the model, the chemical nitrite oxidation rate increases with increasing HNO_2 and DO concentrations in the reactor (**Figure S14**).

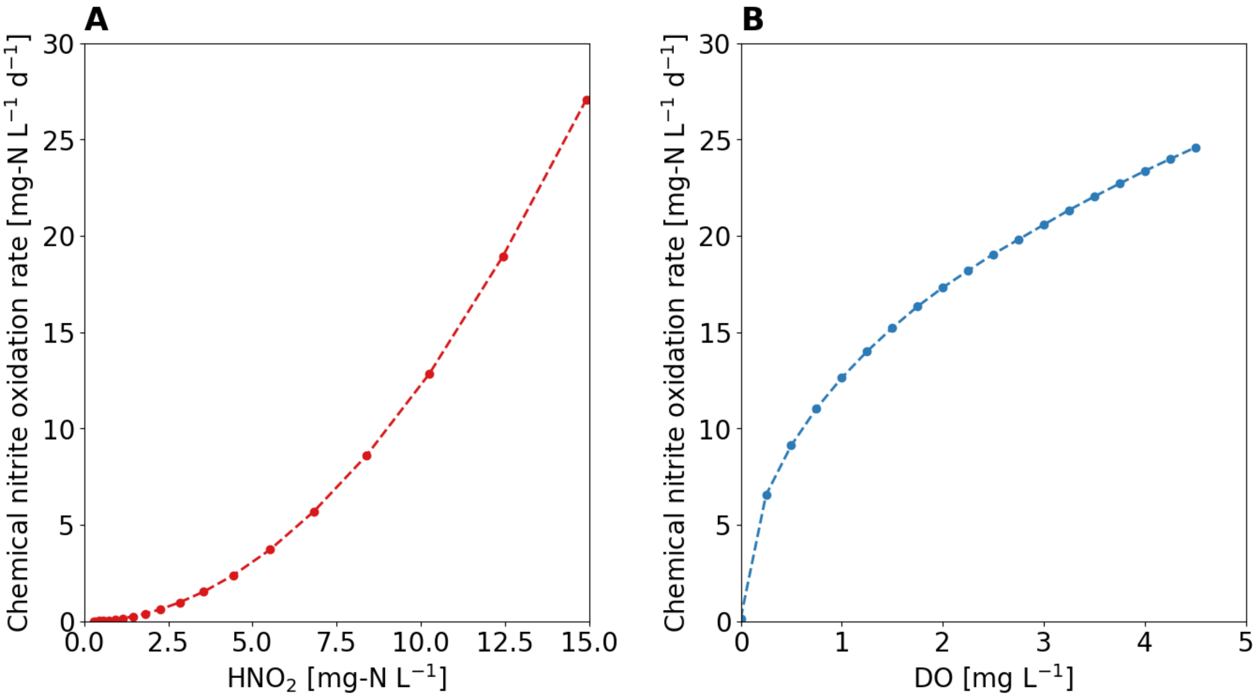

Figure S14: (A) Influence of the HNO_2 concentration on the (simulated) chemical nitrite oxidation given a DO concentration of 5.5 mg L^{-1}. (B) Influence of the DO concentration on the (simulated) chemical nitrite oxidation given a HNO_2 concentration of 15 mg-N L^{-1}

14 Relative abundance of main AOB

Relative abundance of identified AOB species based on 16S rRNA gene-based amplicon sequencing (**Figure S15**). OTU 38 had a 99.06% gene identity to *Nitrosomonas europaea* and OTU 50 had a 99.53% gene identity to "*Candidatus (Ca.)* Nitrosacidococcus urinae".

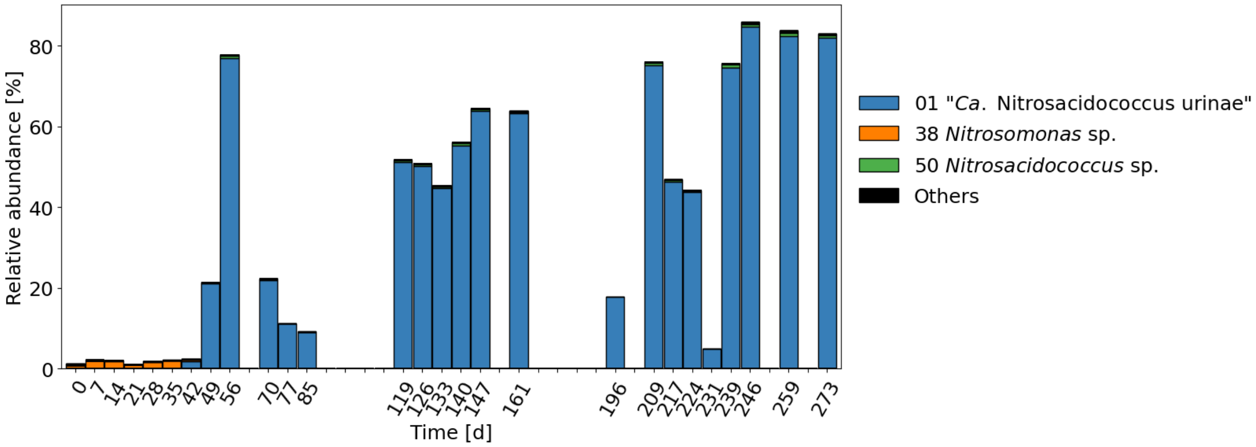

Figure S15: Relative abundance of main AOB species in the main enrichment reactor.

15 Phylogenetic tree "*Ca.* Nitrosacidococcus urinae"

The evolutionary history was inferred using the Neighbor-Joining method (**Figure S16**). The optimal tree with the sum of branch length = 0.19954641 is shown. The tree is drawn to scale, with branch lengths in the same units as those of the evolutionary distances used to infer the phylogenetic tree. The evolutionary distances were computed using the Jukes-Cantor method and are in the units of the number of base substitutions per site. The analysis involved 32 nucleotide sequences. All ambiguous positions were removed for each sequence pair. There were a total of 1541 positions in the final dataset. Evolutionary analyses were conducted in MEGA7. The 16S rRNA identities of "*Ca*. Nitrosacidococcus urinae" with "*Ca.* Nitrosacidococcus tergens" and "*Ca.* Nitrosoglobus terrae" were 99.3% and 92.5%, respectively.

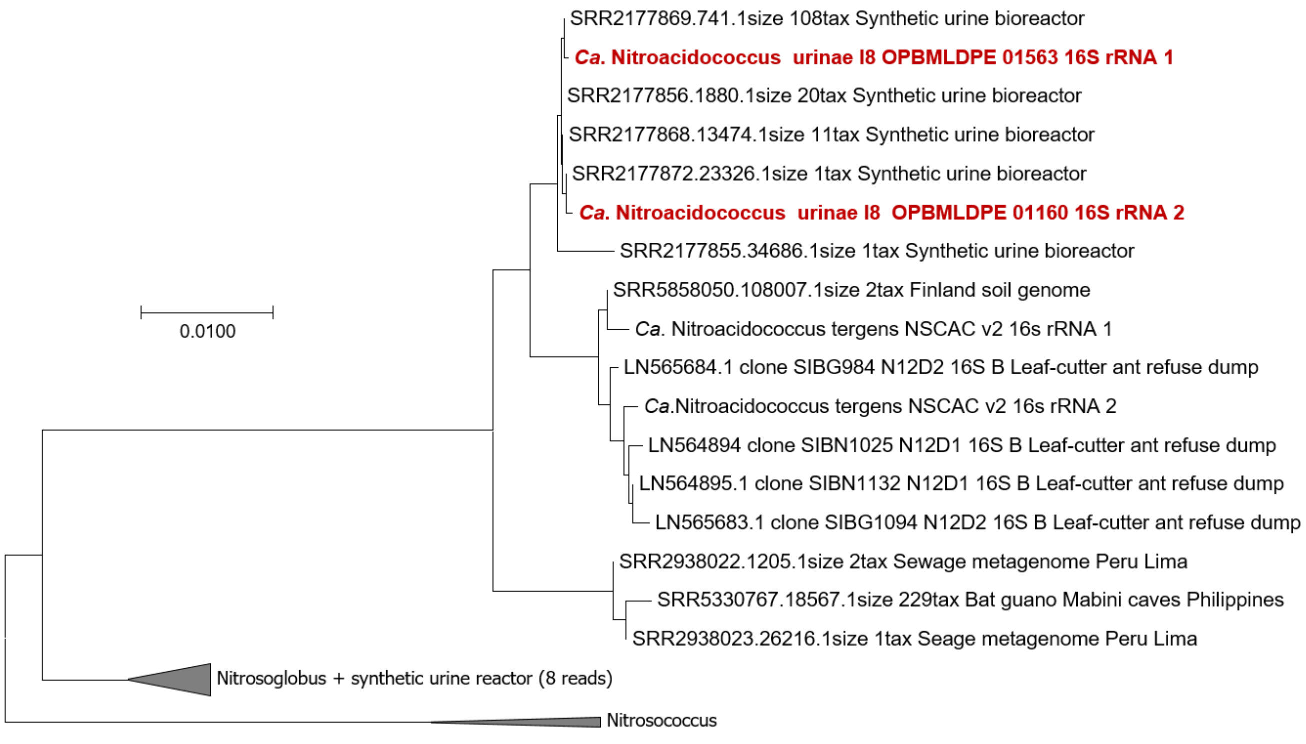

Figure S16: Phylogenetic tree of "*Candidatus* Nitrosacidococcus urinae" I8.

16 Phylogenetic tree and relative abundance of potential NOB

The evolutionary history was inferred using the Neighbor-Joining method (**Figure S17**). The evolutionary distances were computed using the Maximum Composite Likelihood method and are in the units of the number of base substitutions per site. Evolutionary analyses were conducted in MEGA11. While OTU 06 *Xanthobacteraceae* unclassified had a high identity with *Nitrobacter* sp. 219, none of the *Nitrobacter* and *Xanthobacteraceae* linked OTU's actually clustered with known NOB. Regardless, the relative abundance of all *Nitrobacter* and *Xanthobacteraceae* linked OTU that were considered potential NOB decreased below 0.1% after 42 days (**Figure S18**).

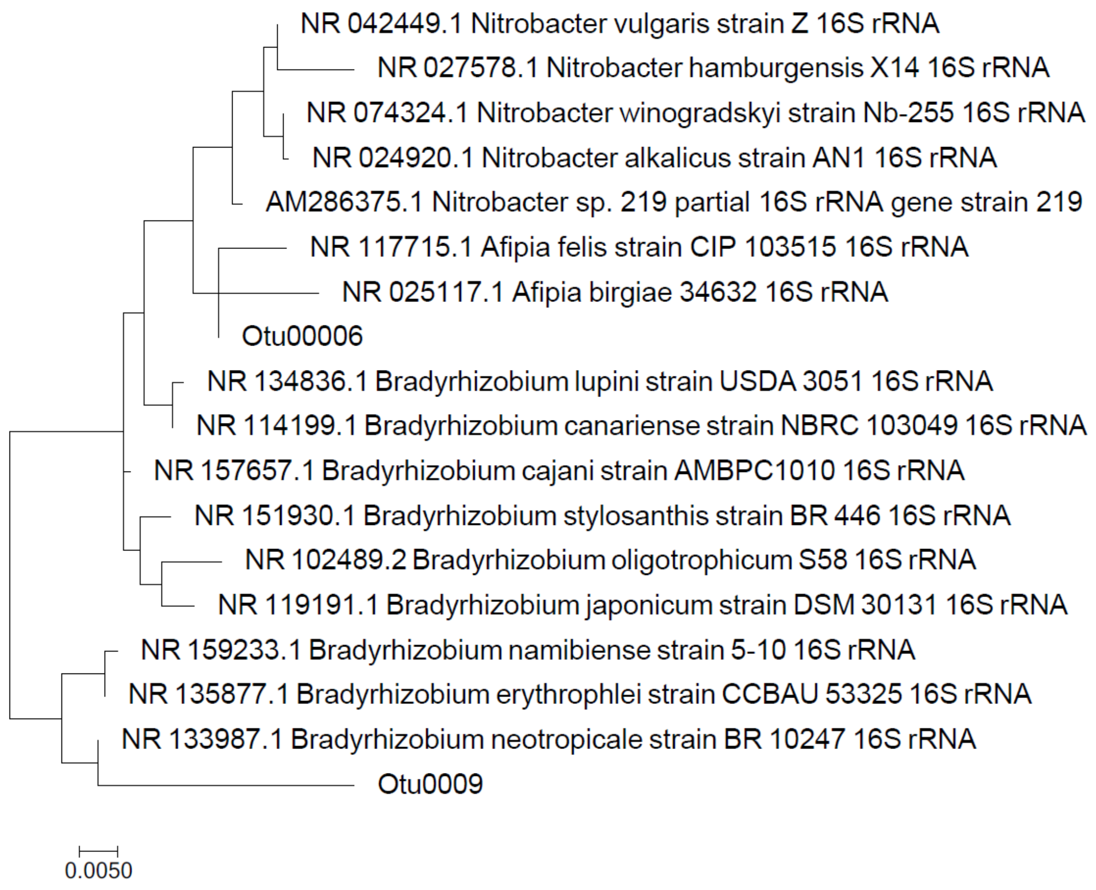

Figure S17: Phylogenetic tree of OTU 06 *Xanthobacteraceae* unclassified and OTU 09 *Xanthobacteraceae* unclassified.

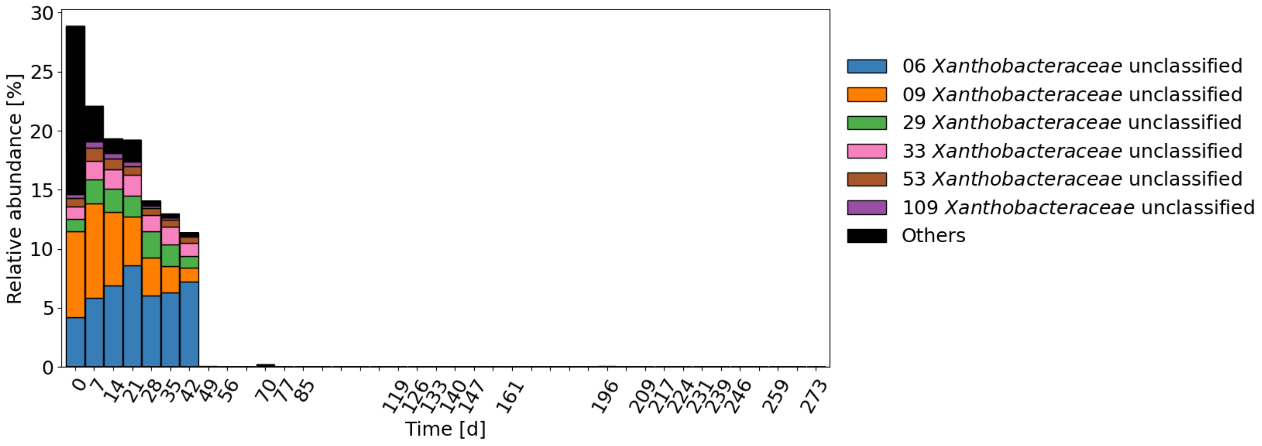

Figure S18: Relative abundance of OUT's associated with *Nitrobacter* and *Xanthobacteraceae* in the main enrichment reactor.

17 Microbial diversity

The shift in the microbial community in general around day 42 was also evident from the reduced diversity in terms of Shannon and inversed Simpson-indices (**Figure S19**), which may be related to selective conditions at pH 5 and the use of COD pretreated influent.

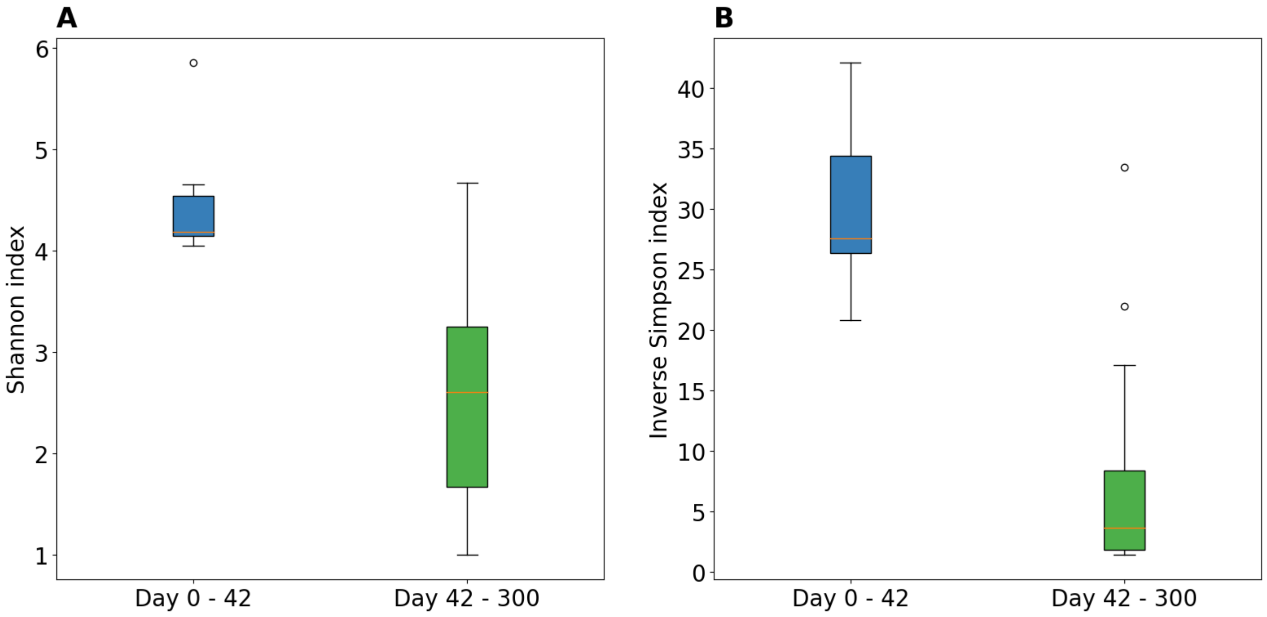

Figure S19: Microbial diversity in the main enrichment reactor before and after day 42. (A) Shannon index. (B) Inverse Simpson index.

18 Particle size distribution

The particle size distribution showed that no large aggregates (diameter > 100 µm) were formed (**Figure S20**). Therefore, pH adaption mechanism with the help of pH-neutral micro-environments such as granules or biofilm, as described in De Boer and Kowalchuk (2001), can be excluded in the present system.

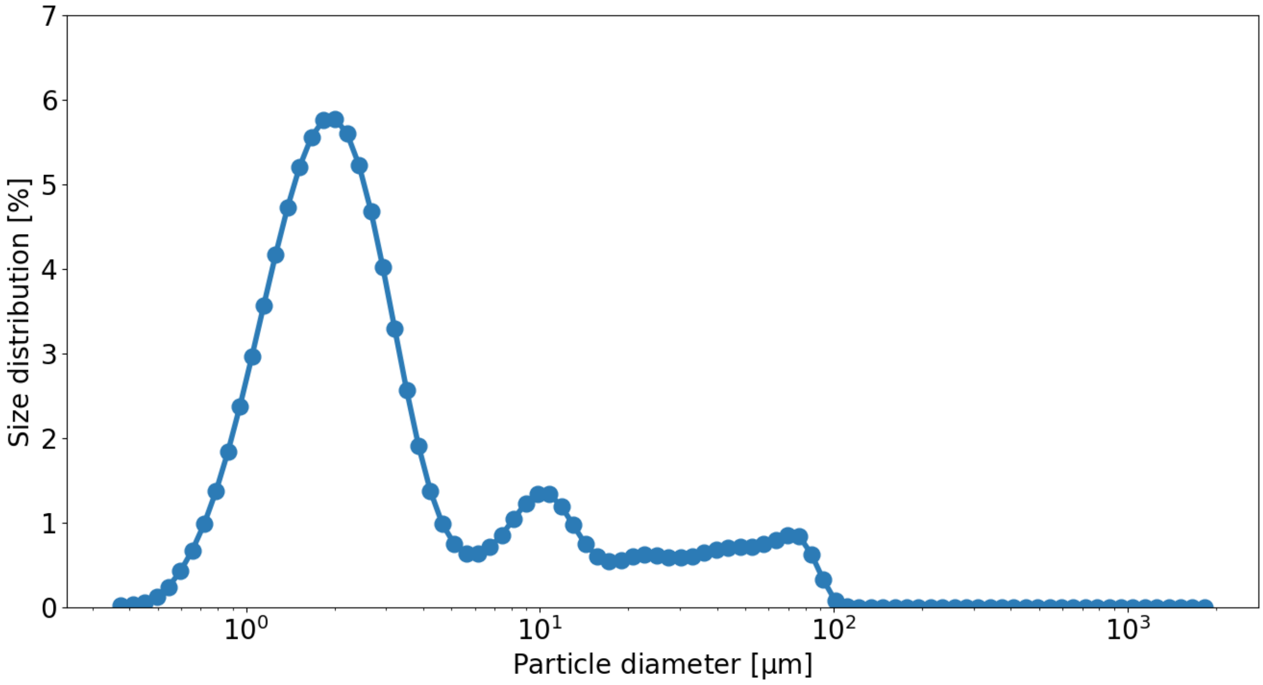

Figure S20: Particle size distribution in the acidic enrichment reactor and in a urine nitrification reactor operated at pH 6 (120-L urine nitrification reactor at Forum Chriesbach, Eawag, Switzerland).

19 Net growth rate

Since there is no sludge retention, the hydraulic retention time (HRT) was equal to the solid retention time (SRT). **Figure S21A** shows the SRT. Since the flow rate and thus the SRT is determined by the ammonia oxidation rate, the SRT decreased with increasing ammonia oxidation rate increased, but never fell below 1.6 days indicating the minimal required SRT for the growth of the acid-tolerant AOB at pH 5. This SRT corresponds to a maximum net growth rate of around 0.6 d^{-1} (see also **Figure S21B**).

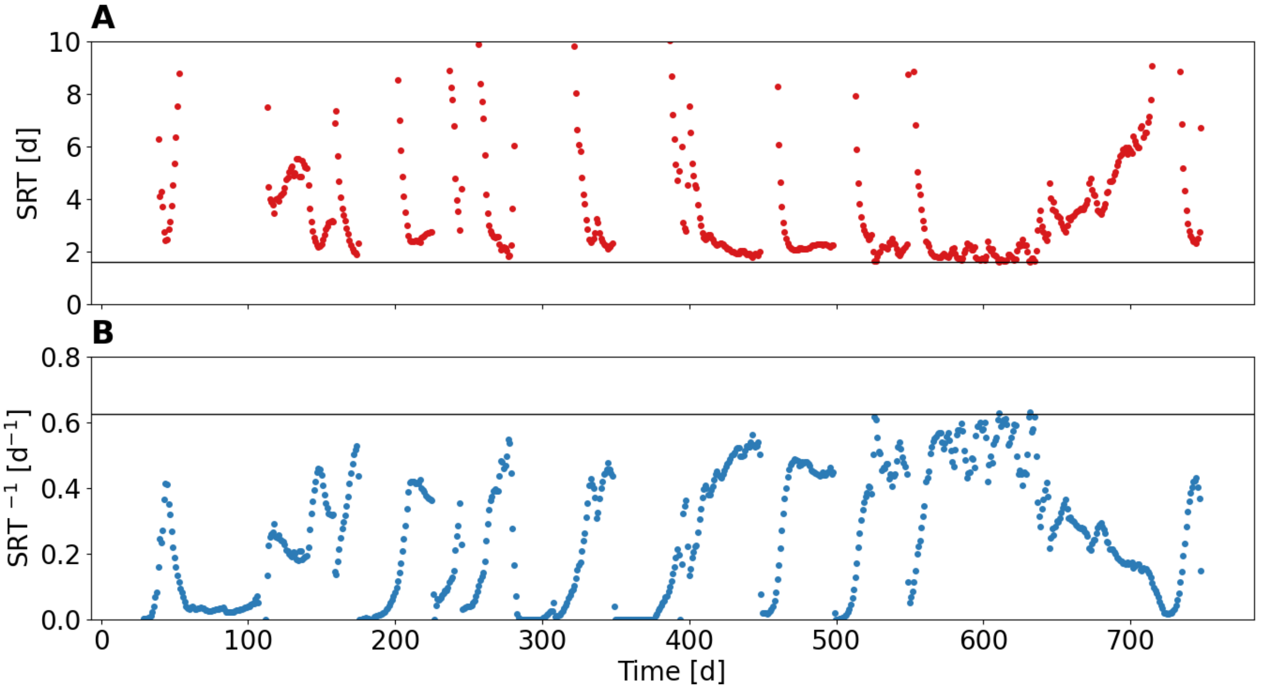

Figure S21: (A) Solid retention time (SRT). (B) Inverse of the SRT as a proxy of the net growth rate during quasi steady-state conditions.

20 Influence of anoxic conditions (no aeration)

The chemical nitrite oxidation model in SUMO was used to simulate the NO concentration during anoxic phases at pH 5 and 7 (**Figure S22**). Due to chemical nitrite oxidation, the NO concentration increased during non-aerated phases at low pH values.

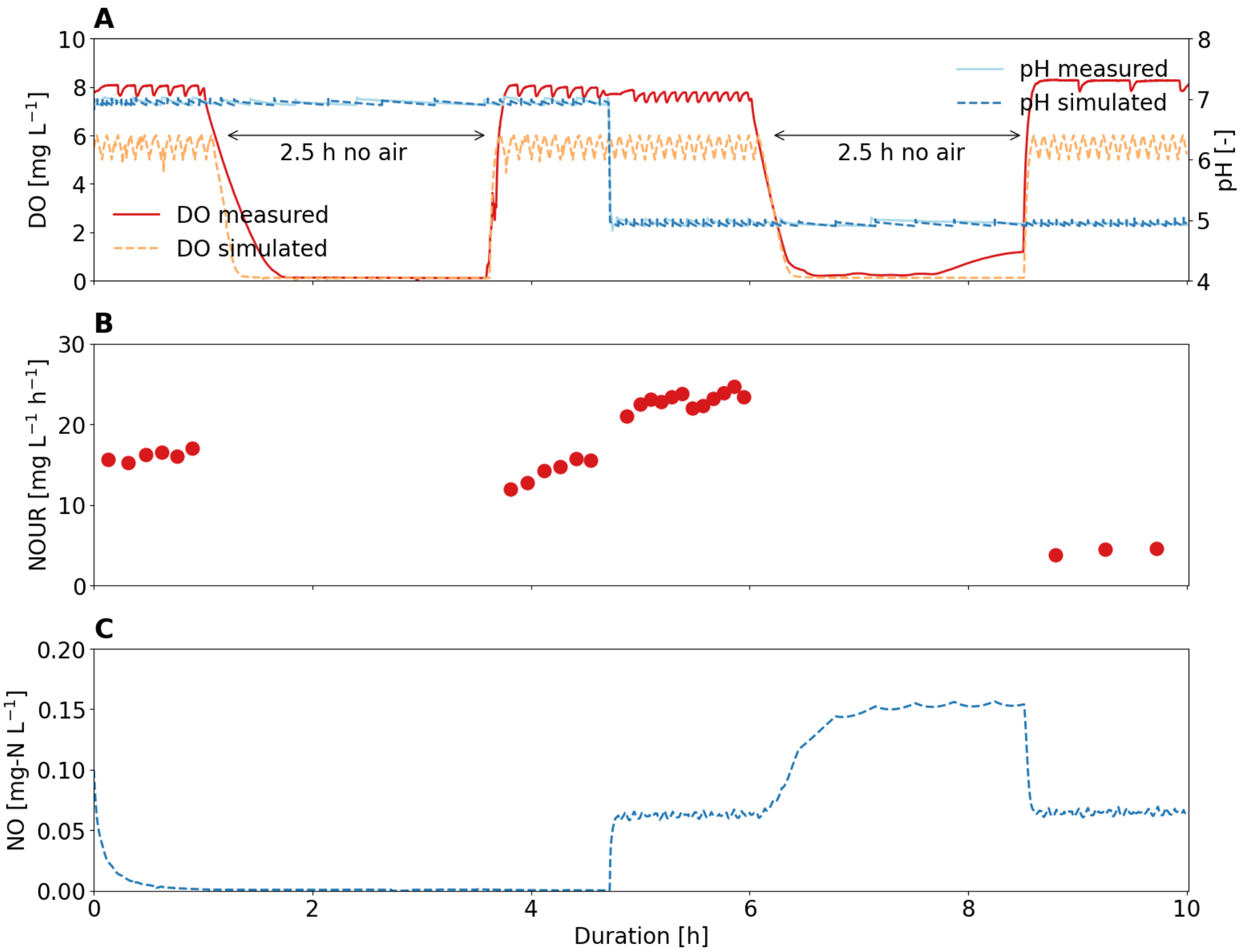

Figure S22: Short-term experiment to study the effect of anoxic phases at pH 5 and 7. The TNN concentration during the experiment was about 500 mg-N L^{-1}. (A) Measured and simulated dissolved oxygen (DO) concentration and pH. (B) Measured nitrogenous oxygen uptake rate (NOUR). (C) Simulated dissolved NO concentration.

21 Influence of anoxic conditions (N_2 stripping)

The chemical nitrite oxidation model in SUMO was used to simulate the NO concentration during a one-hour long phase at pH 5 in which aeration was turned off and N_2 was injected instead (**Figure S23**). Due to the N_2 stripping, the NO concentration increased only slightly.

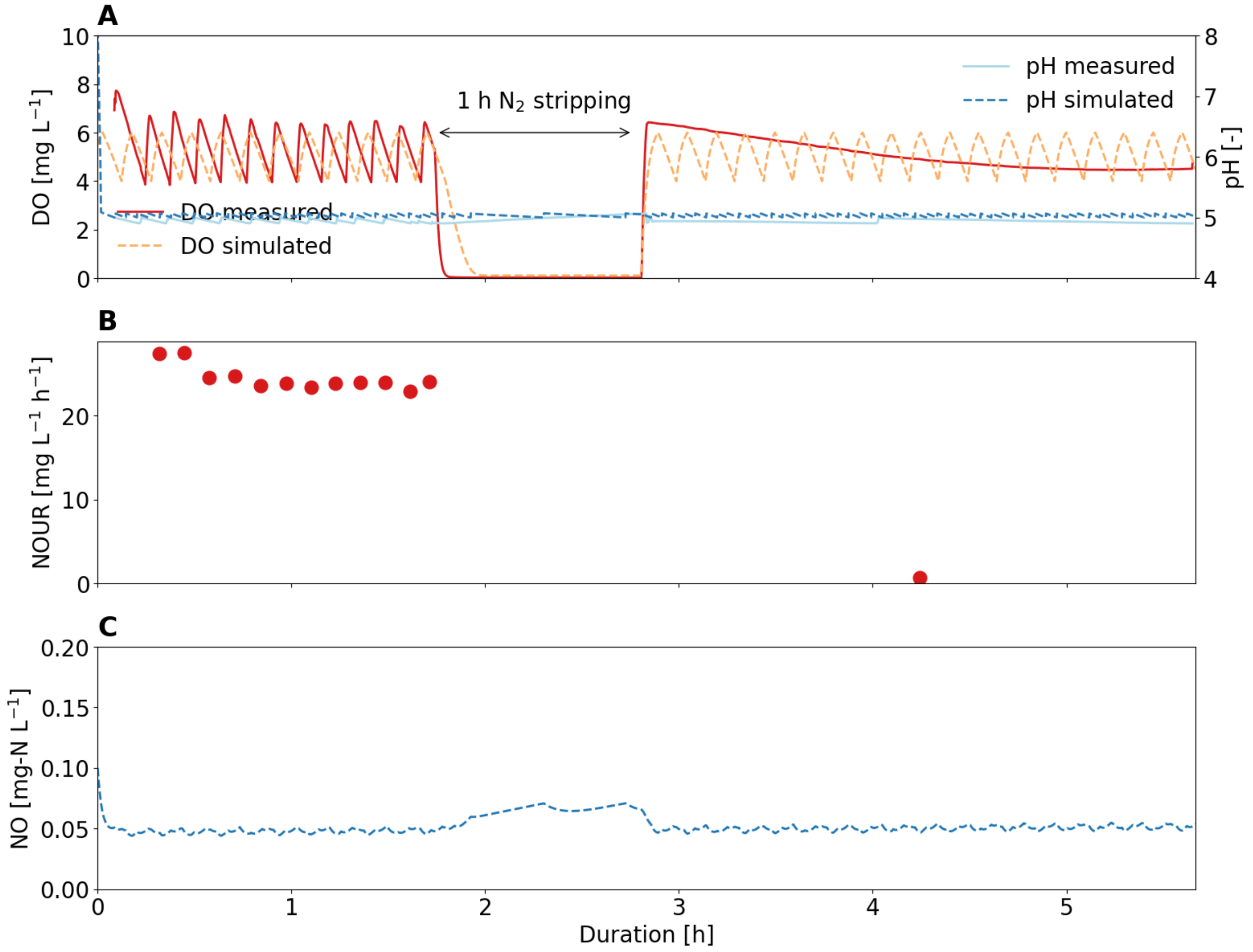

Figure S23: Short-term experiment to study the effect of anoxic phases at pH 5 and 7. The TNN concentration during the experiment was about 500 mg-N L^{-1}. (A) Measured and simulated dissolved oxygen (DO) concentration and pH. (B) Measured nitrogenous oxygen uptake rate (NOUR). (C) Simulated dissolved NO concentration.

22 Batch activity tests without pH control

Batch experiments without pH control were performed using activated sludge from the main enrichment reactor to which either nitrite or ammonium was added to achieve different concentrations of TAN and TNN (**Figure S24**). The different TAN concentrations of 850 mg-N L^{-1} and 1700 mg-N L^{-1} did not affect the pH at which the ammonia oxidation ceased. However, the different TNN concentrations of 350 mg-N L^{-1}, 700 mg-N L^{-1}, and 1200 mg-N L^{-1} affected the minimum pH values. This suggests that the cessation of ammonia oxidation at low pH is most likely not related to NH_3 or a direct pH effect, but rather to the HNO_2 concentration or a substance which is proportional to HNO_2, such as NO.

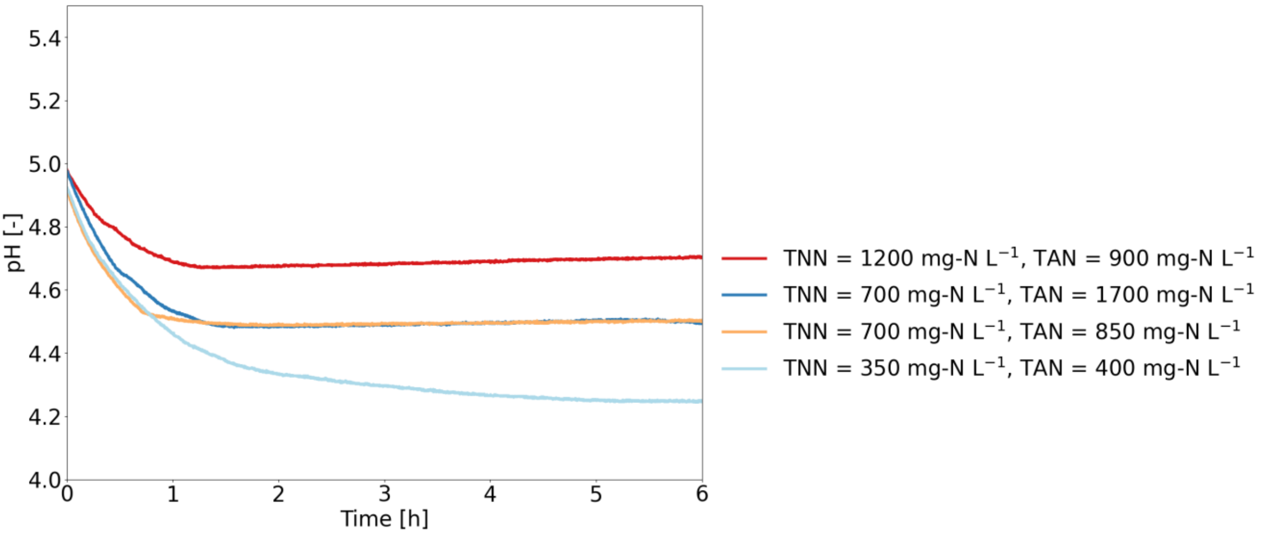

Figure S24: Batch activity tests without pH control.

23 NO concentration main reactor

The NO concentration in the reactor was simulated using the chemical nitrite oxidation model in SUMO (**Figure S25**). The two operational disturbances that led to an influent stop and a pH decrease after 159 days and 226 days resulted in an increased concentration of dissolved NO in the reactor.

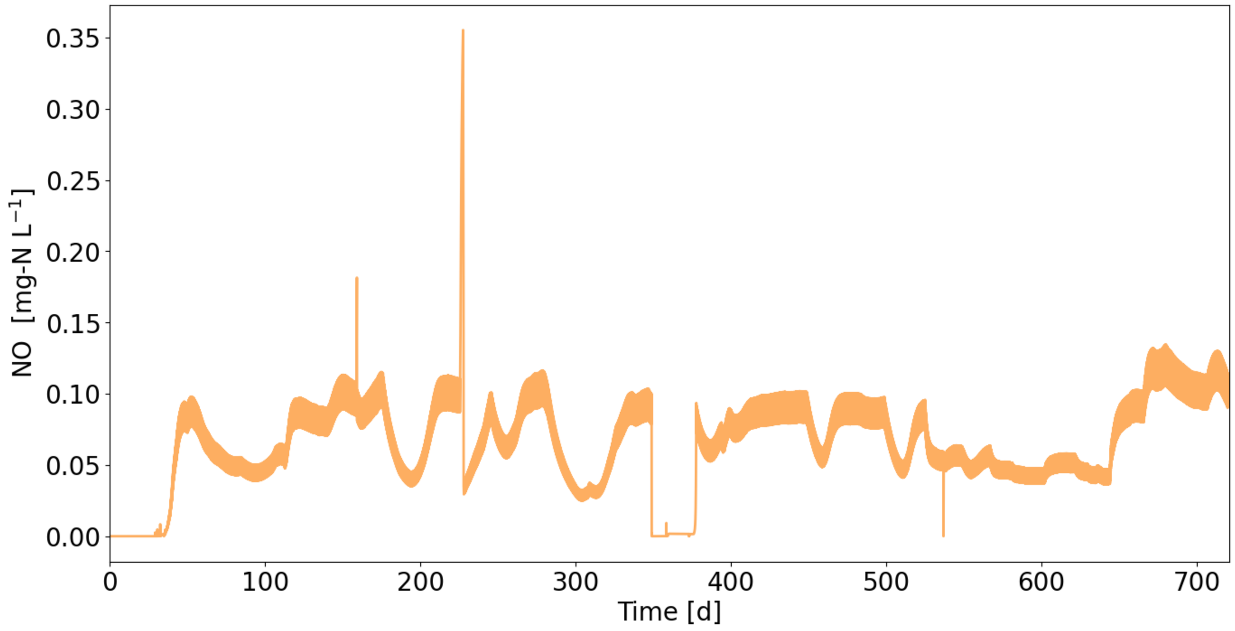

Figure S25: Simulated NO concentration in the main enrichment reactor. The peaks in NO after 159 days and 226 days were due to the stop of the influent causing a pH drop and an increased HNO_2 concentration.

24 Salinity

At values above 20 mS cm^{-1}, salinity had an inhibitory effect on the activity of acid-tolerant AOB, which is consistent with what was found for acid-sensitive AOB in urine treatment (**Figure S26**).

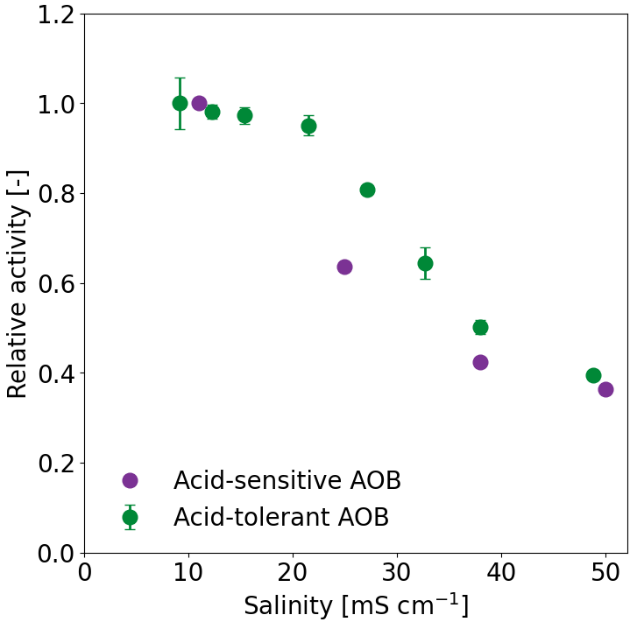

Figure S26: Influence of salinity on the activity of activated sludge dominated by the acid-tolerant AOB "*Ca.* Nitrosacidococcus urinae" I8 and acid-sensitive AOB of the *Nitrosomonas europaea* lineage. The activity is expressed as relative activity by dividing the nitrogenous oxygen uptake rate (NOUR) through the NOUR without changes.

25 Influence of dissolved oxygen substrate limitation

The DO affinity constant for the acid-tolerant AOB was estimated to be approximately 0.8 mg L^{-1} (**Figure S27**).

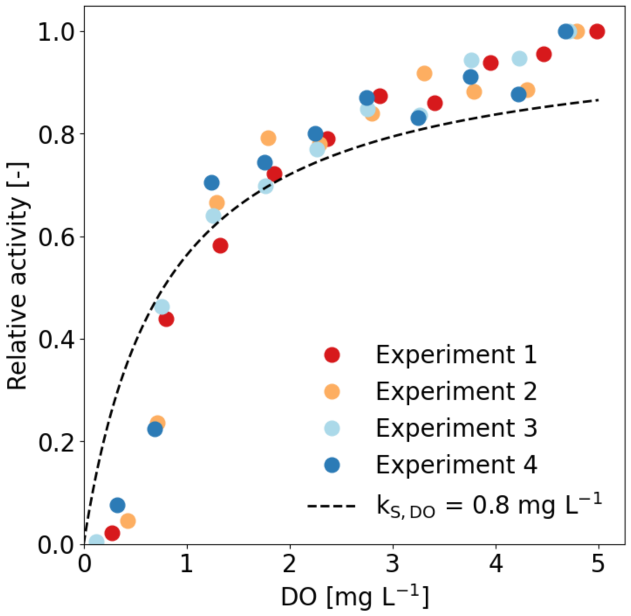

Figure S27: Influence of dissolved oxygen (DO) concentrations on the activity of activated sludge dominated by "*Ca.* Nitrosacidococcus urinae" I8. The activity is expressed as relative activity by dividing the nitrogenous oxygen uptake rate (NOUR) b they NOUR at a DO of 5 mg L^{-1}.

26 Phylogenetic tree of OTU 16, 51, and 94

The evolutionary history was inferred using the Neighbor-Joining method (**Figure S28**). The evolutionary distances were computed using the Maximum Composite Likelihood method and are in the units of the number of base substitutions per site. This analysis involved 27 nucleotide sequences. All ambiguous positions were removed for each sequence pair (pairwise deletion option). There were a total of 426 positions in the final dataset. Evolutionary analyses were conducted in MEGA11.

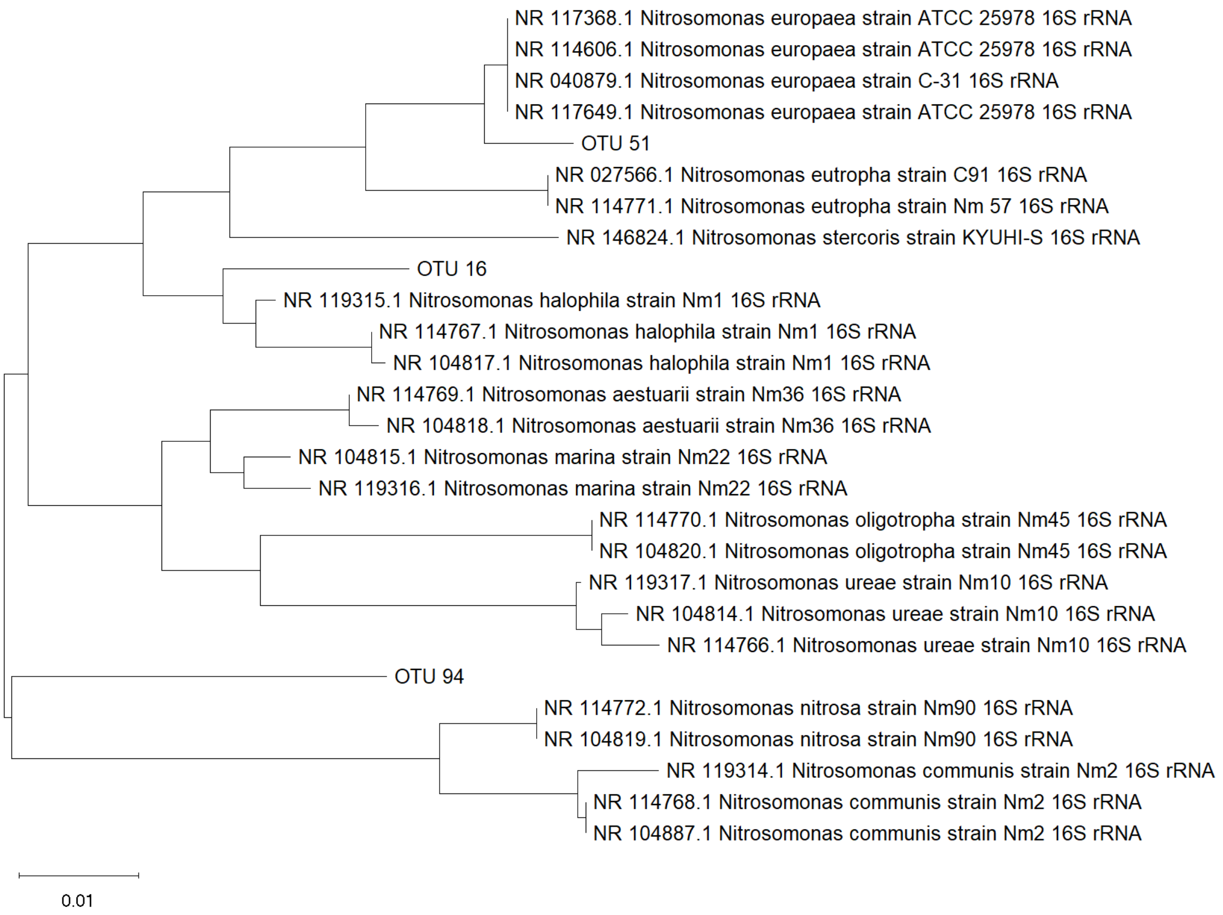

Figure 28: Phylogenetic tree of OTU 16 *Nitrosomonas* sp., OTU 51 *Nitrosomonas* sp., and OTU 94 *Nitrosomonas* sp. OTU 16 clustered with the *Nitrosomonas halophila* lineage and OTU 51 with the *Nitrosomonas europaea* lineage. OTU 94 seems to belong to the *Nitrosomonas* group but is not closely related to any known *Nitrosomonas* species

27 pH drop experiments

To test whether acid-tolerant AOB were still abundant, activated sludge was removed from the 12-L reactor, washed with nitrified urine, and added to an aerobic batch reactor without influent and pH control (**Figure S29**). A pH decrease well below 5.4 indicated the presence of acid-tolerant AOB. Until day 311, the pH decreased below 5.4, indicating the abundance of acid-tolerant AOB. The activated sludge from day 364 and day 385 did not decrease below 5.4 anymore, indicating that acid-tolerant AOB were no longer present in the AS.

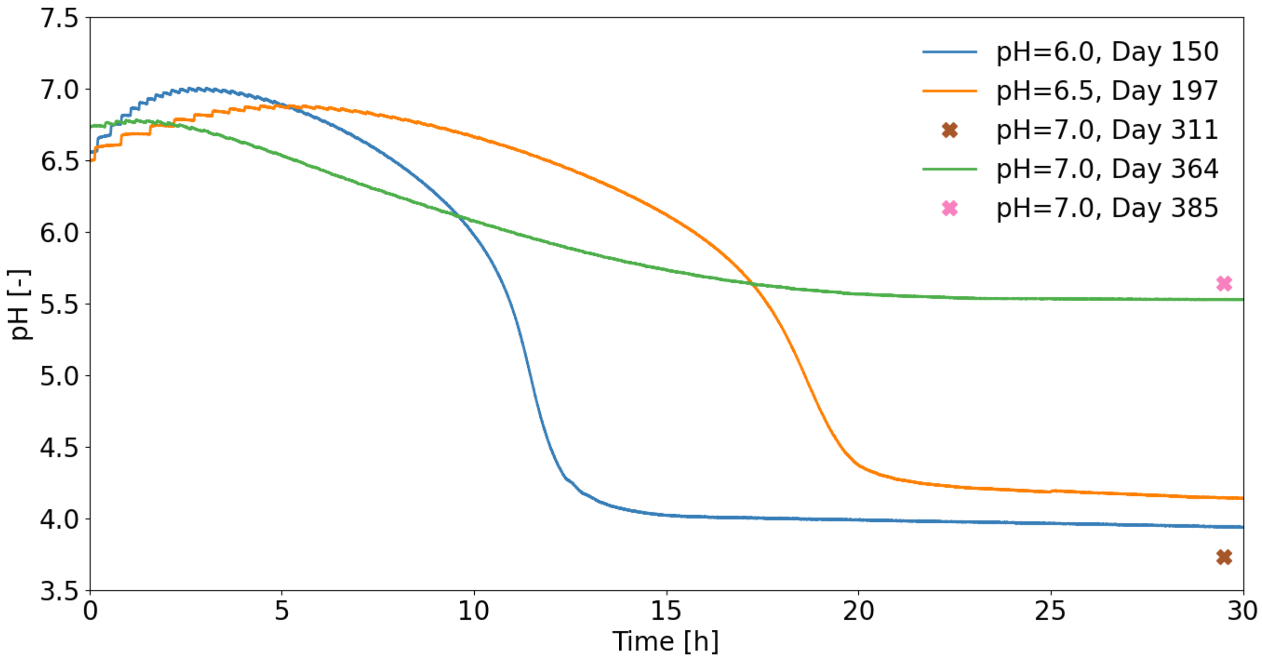

Figure S29: pH batch experiments with washed activated sludge from different time periods of the long-term pH experiment. For the AS on day 311 and day 385, only the pH value at the end of the experiment was measured.

28 NOB pH long-term experiment

As in the main enrichment reactor, the relative abundance of all *Nitrobacter* and *Xanthobacteraceae* linked OTU, which were considered potential NOB, decreased below 0.1% after the pH was increased to 6 after 130 days. (**Figure S30**).

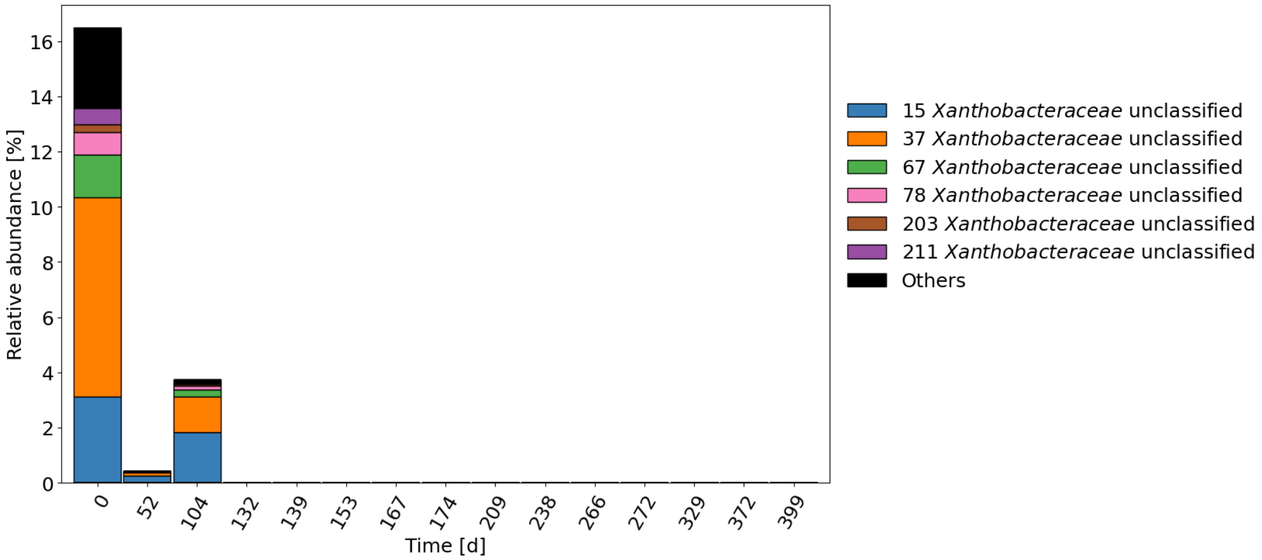

Figure S30: Relative abundance of top 6 OUTs associated with *Nitrobacter* and *Xanthobacteraceae* in the long-term pH experiment.

29 Net growth rate long-term pH experiment

Figure S31 shows the inverse of the solid retention time (SRT), which would corresponds to the net growth rate at steady state. While maximum net growth rates around 0.6 d^{-1} were observed between pH 5 to 6.5, the net growth rate drops to 0.1 d^{-1} at pH 7 and probably increases only when the community switched from the acid-tolerant AOB *"Ca.* Nitrosacidococcus urinae" to the acid-sensitive *Nitrosomonas* lineage.

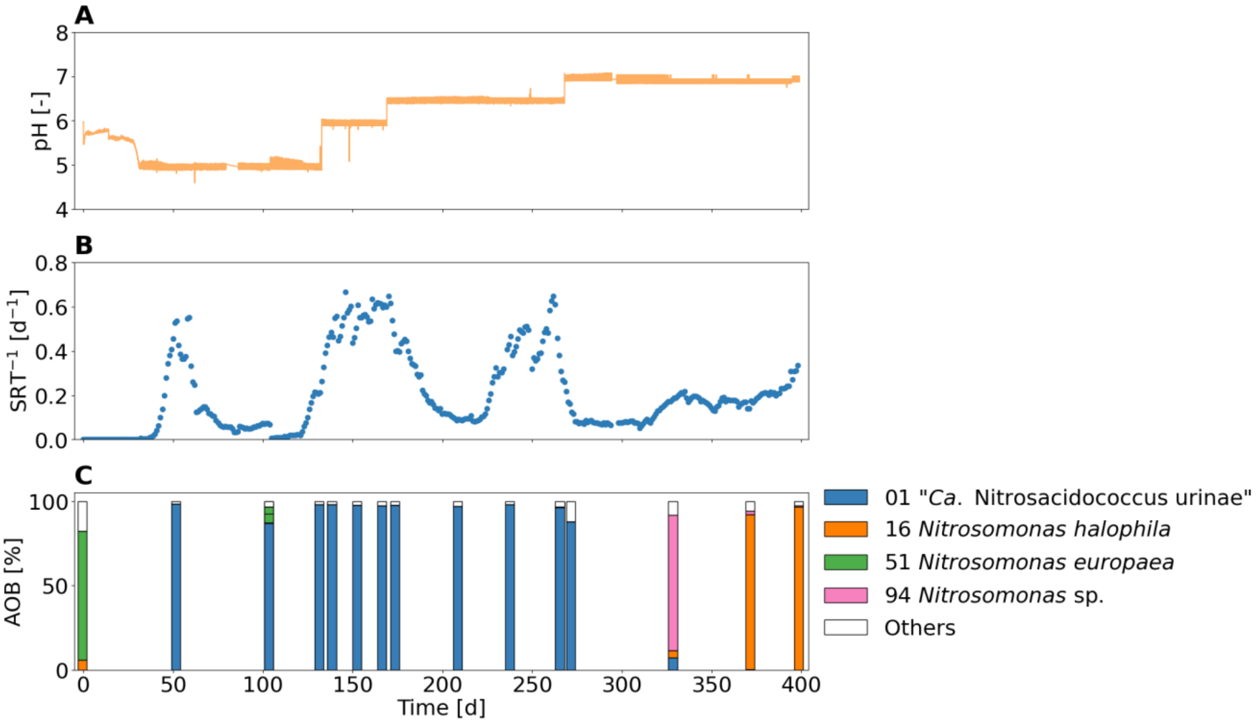

Figure S31: Performance of the ammonia oxidation reactor operated for 400 days at pH values between 5 and 7. (A) pH. (B) Inverse of the solid retention time (SRT) (C) Relative abundance of AOB species compared to all recognized AOB species.

30 PHREEQC simulation of dissolved iron (Fe^{2+}, Fe^{3+}) and copper (Cu^{2+}, Cu^{+})

Dissolved iron (Fe^{3+} and Fe^{2+}) is more abundant at low pH due to the iron complexation equilibrium (**Figure S32A**), as low pH minimized chemical iron oxidation and maximizes iron solubility (Ferguson and Ingledew 2008). On the other hand, the influence of pH on copper, which is another important trace element for AOB, is much lower (**Figure S32B**).

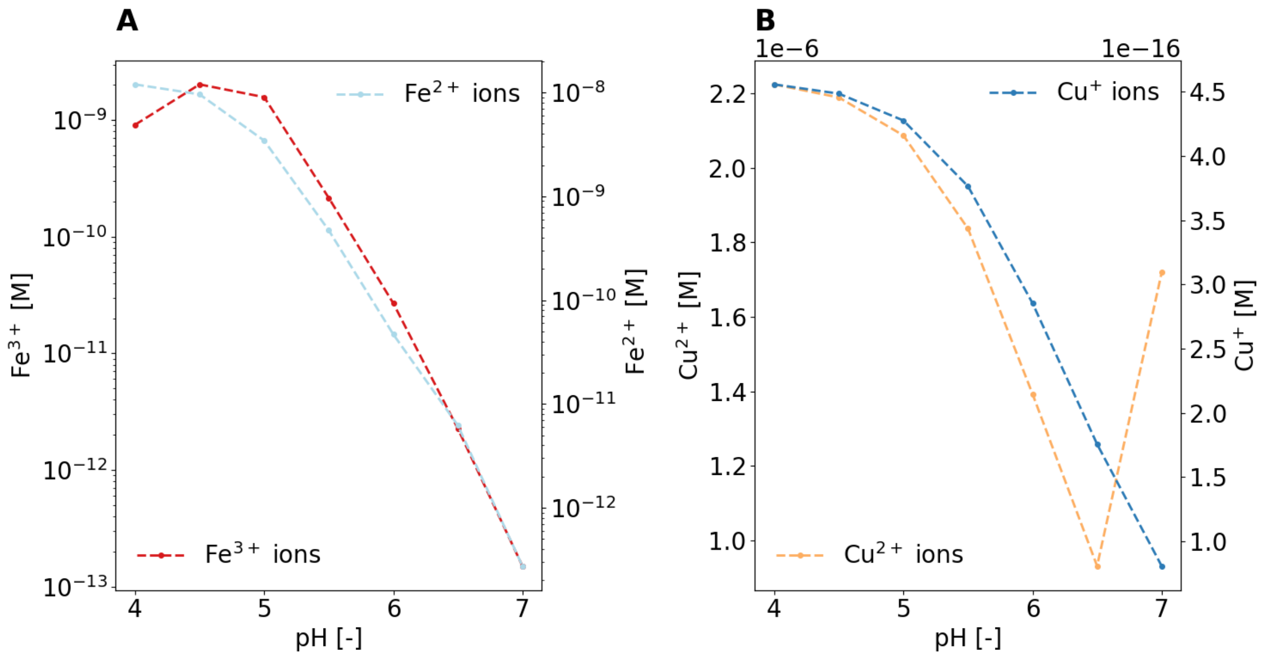

Figure S32: (A) Modeling of Fe^{3+} and Fe^{2+} in urine with PHREEQC. The total iron concentration for the simulation was 100 µg L^{-1}. The y-axes are given in a logarithmic scale. (B) Modeling of Cu^{2+} and Cu^{+} in urine with PHREEQC. The total copper concentration for the simulation was 190 µg L^{-1}.

31 Iron dosage long-term pH experiment – batch experiment

The batch test with washed activated sludge from day 51 indicates that acid-tolerant AOB were still active at pH 7 and high iron concentration (**Figure S33**).

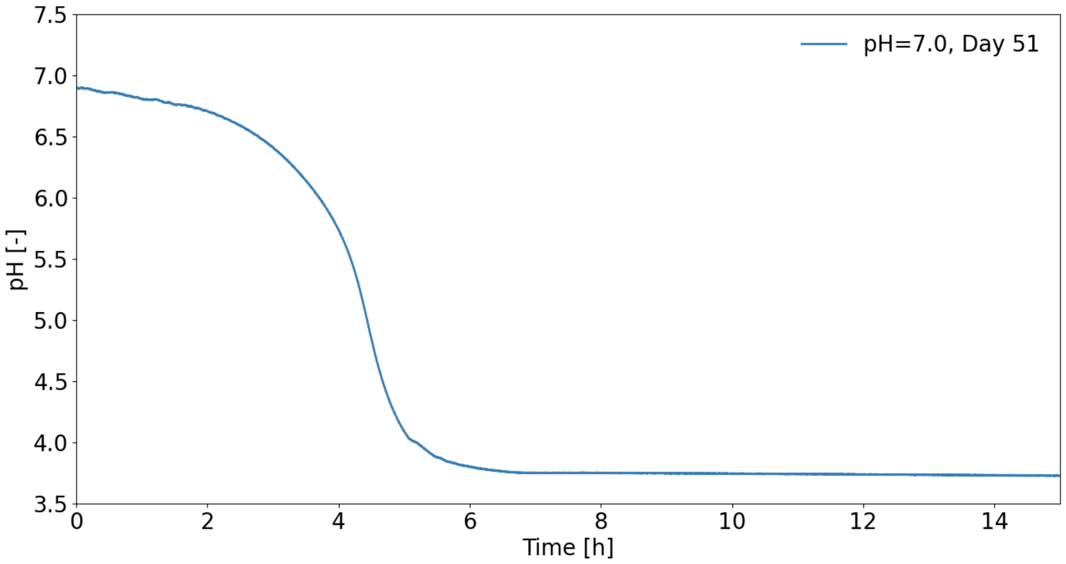

Figure S33: pH batch test with washed activated sludge from day 51 of the long-term iron experiment.

32 Iron dosage long-term pH experiment - dilution rate

Figure S34 shows the inverse of the solid retention time (SRT), which would corresponds with the net growth rate at steady state. After increasing the pH to 7 and adding iron, the inverse of the SRT increased up to 1 d^{-1}, which is higher than ever observed before. Only after the influent was replaced did the inverse of the SRT decrease to about 0.1 d^{-1}.

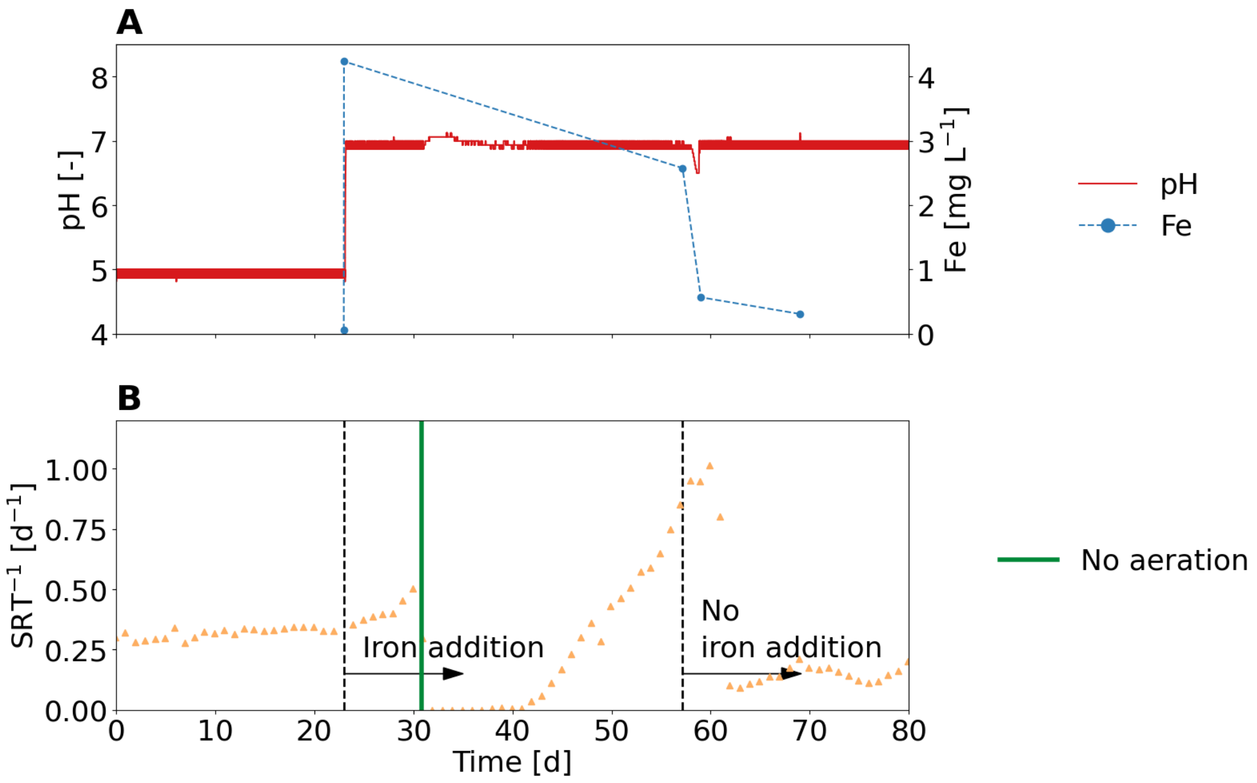

Figure S34: Performance of the ammonia oxidation reactor operated for 70 days at pH 5 and 7 and spiked with iron. (A) pH and total iron (Fe) concentrations in the reactor. After 22 days, pH was increased and iron was added to the reactor and the influent. After 57 days, influent without iron addition was used (B) Inverse of solid retention time (SRT) and operational disturbance.

33 Concentration of trace elements

Iron (Fe), copper (Cu), cobalt (Co), nickel (Ni), aluminum (Al), manganese (Mn), zinc (Zn), cadmium (Cd), and lead (Pb) were measured in the influent (**Table S10**) and reactor (**Table S11**) using inductively coupled plasma mass spectrometry.

Table S10: Concentration of trace elements in the influent. *After 22 days, the pH was increased and iron and NTA was added to the reactor and influent. ⁺After 57 days, influent without iron addition was used.

**Time [d]**	**Fe [µg L^{-1}]**	**Cu [µg L^{-1}]**	**Co [µg L^{-1}]**	**Ni [µg L^{-1}]**	**Al [µg L^{-1}]**	**Mn [µg L^{-1}]**	**Zn [µg L^{-1}]**	**Cd [µg L^{-1}]**	**Pb [µg L^{-1}]**	**Cr [µg L^{-1}]**
22	74	41	0.07	8.6	5.9	2.4	78	0.01	0.23	6.7
28*	3607	25	0.14	8.1	9.8	16.4	73	0.01	0.12	12.3
56	3358	61	0.19	14.6	10.9	15.6	121	0.02	0.80	4.8
57^{+}	354	63	0.09	12.4	6.4	1.9	136	0.02	2.21	3.0
69	436	75	0.18	12.0	39.3	3.3	179	0.03	1.15	9.3

Table S11: Concentration of trace elements in the reactor. *After 22 days, the pH was increased and iron and NTA was added to the reactor and influent. ⁺After 57 days, influent without iron addition was used.

**Time [d]**	**Fe [µg L^{-1}]**	**Cu [µg L^{-1}]**	**Co [µg L^{-1}]**	**Ni [µg L^{-1}]**	**Al [µg L^{-1}]**	**Mn [µg L^{-1}]**	**Zn [µg L^{-1}]**	**Cd [µg L^{-1}]**	**Pb [µg L^{-1}]**	**Cr [µg L^{-1}]**
22	61	62	0.52	29.4	8.3	3.7	261	0.04	5.45	12.2
23*	4239	67	1.33	32.7	10.3	14.0	182	0.03	5.05	11.1
57	2578	59	0.31	25.1	9.2	13.3	152	0.02	1.69	4.6
59^{+}	568	55	0.20	21.7	6.9	4.1	141	0.02	1.80	3.6
69	310	108	0.5	21.6	20.8	5.2	136	0.03	1.38	4.8

34 References

Anthonisen, A.C., Loehr, R.C., Prakasam, T.B.S. and Srinath, E.G. (1976) Inhibition of Nitrification by Ammonia and Nitrous-Acid. Journal Water Pollution Control Federation 48(5), 835-852.

Crittenden, J.C., Trussell, R.R., Hand, D.W., Kerry, J.H. and Tchobanoglous, G. (2012) MWH's Water Treatment: Principles and Design, John Wiley & Sons, New Jersey, USA.

Davies, C.W. (1967) Electrochemistry, Philosophical Library, London.

De Boer, W. and Kowalchuk, G.A. (2001) Nitrification in acid soils: micro-organisms and mechanisms. Soil Biology & Biochemistry 33(7-8), 853-866.

Ferguson, S.J. and Ingledew, W.J. (2008) Energetic problems faced by micro-organisms growing or surviving on parsimonious energy sources and at acidic pH: I. Acidithiobacillus ferrooxidans as a paradigm. Biochim Biophys Acta 1777(12), 1471-1479.

Fumasoli, A., Burgmann, H., Weissbrodt, D.G., Wells, G.F., Beck, K., Mohn, J., Morgenroth, E. and Udert, K.M. (2017) Growth of Nitrosococcus-Related Ammonia Oxidizing Bacteria Coincides with Extremely Low pH Values in Wastewater with High Ammonia Content. Environmental Science & Technology 51(12), 6857-6866.

Levine, I.N. (1988) Physical Chemistry, McGraw-Hill, New York.

Lewis, G.N. and Randall, M. (1921) The activity coefficient of strong electrolytes. Journal of the American Chemical Society 43(5), 1112-1154.

Lide, D.R. (2009) CRC Handbook of Chemistry and Physics, CRC Press/Taylor and Francis, Boca Raton, FL.

Stumm, W. and Morgan, J.J. (1996) Aquatic Chemistry: Chemical Equilibria and Rates in Natural Waters, Wiley.

Udert, K.M., Larsen, T.A. and Gujer, W. (2005) Chemical nitrite oxidation in acid solutions as a consequence of microbial ammonium oxidation. Environmental Science & Technology 39(11), 4066-4075.

Zacharia, I.G. and Deen, W.M. (2005) Diffusivity and solubility of nitric oxide in water and saline. Ann Biomed Eng 33(2), 214-222.
